# Improved Air Stability of Li Argyrodites Through PS_4_^3−^ Rotation Suppression by Al and Se Co‐Substitution for All‐Solid‐State Batteries

**DOI:** 10.1002/advs.202519093

**Published:** 2025-12-16

**Authors:** Juhyoun Park, Jihun Lee, Yoon‐Seong Kim, Donghyeok Kim, Minseo Jang, Junwoo Lee, Hae‐Yong Kim, Changhun Park, Jeongheon Kim, Habin Chung, Kyung‐Wan Nam, Dong‐Hwa Seo, Yoon Seok Jung

**Affiliations:** ^1^ Department of Chemical and Biomolecular Engineering Yonsei University Seoul 03722 Republic of Korea; ^2^ Department of Battery Engineering Yonsei University Seoul 03722 Republic of Korea; ^3^ Department of Materials Science and Engineering Korea Advanced Institute of Science and Technology (KAIST) Daejeon 34141 Republic of Korea; ^4^ LG Chem, Ltd LG Science Park, 30, Magokjungang 10‐ro, Gangseo‐gu Seoul 07796 Republic of Korea; ^5^ Department of Energy and Materials Engineering Dongguk University 30 Pildong‐ro 1‐gil, Jung‐gu Seoul 04620 Republic of Korea

**Keywords:** air stability, all‐solid‐state batteries, ionic conductivities, sulfide solid electrolytes, surface degradation

## Abstract

Sulfide‐based solid electrolytes, particularly Li argyrodites, hold significant promise for practical all‐solid‐state batteries (ASSBs); however, their poor stability under humid conditions presents a critical challenge. Despite numerous efforts to address this issue, a comprehensive mechanistic understanding of moisture‐induced degradation remains limited. Herein, we introduce an Al and Se co‐substituted argyrodite, Li_6‐3_ *_x_* Al*_x_* PS_5‐1.5_ *_x_* Se_1.5_ *_x_* Cl, which enhances both the Li^+^ conductivity and air stability. The optimized composition (*x* = 0.05) exhibits an improved Li^+^ conductivity of 4.91 mS cm^−1^ at 30 °C and a 22% conductivity reduction after dry‐air exposure (dew point: −40 °C for 5 h), compared with 3.71 mS cm^−1^ and a 42% decrease for the unsubstituted sample. Reduced surface degradation is validated by comprehensive experimental analyses. Complementary calculations indicate less favorable H_2_O adsorption and further reveal that Al and Se co‐substitution inhibits the rotation of P[S_2_SeO]^3−^ and P[S_2_O_2_]^3−;^ tetrahedra via preferential surface‐oriented Se^2−^ and Al─O interactions, which otherwise promote H_2_O‐induced degradation, thereby minimizing moisture interactions. Finally, the improved electrochemical performance of the co‐substituted argyrodite is validated by its enhanced capacity retention following air exposure in NCM|Li_6_PS_5_Cl|(Li‐In) cells. This study highlights rotational dynamics as an overlooked mechanism underlying moisture‐induced degradation, and demonstrates that targeted co‐substitution is a viable strategy for advancing practical ASSBs.

## Graphical abstract

Al/Se co‐substitution in Li_6_PS_5_Cl achieves enhanced dry‐air stability and improves Li^+^ conductivity, retaining 85% of its high Li^+^ conductivity (4.91 mS cm^−1^ at 30 °C) after dry‐air exposure (dew point −40 °C for 5 h). The stability enhancement is experimentally validated and underpinned by theoretical analyses indicating suppressed rotation of P[S_2_SeO]^3−^. Consequently, dry‐air‐exposed Al/Se‐substituted Li_6_PS_5_Cl enables NCM||(Li‐In) cells to deliver superior electrochemical performance.

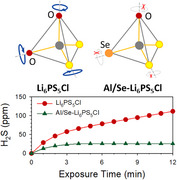

## 1 Introduction

Sulfide‐based solid electrolytes (SEs) offer manufacturing‐friendly mechanical deformability and robust interfacial compatibility with cell components in all‐solid‐state batteries (ASSBs),^[^ ^1^, ^2^, ^3^, ^4^ ^]^ positioning them at the forefront of this field. Among these, highly conductive Li argyrodites, Li_6‐a_PS_5‐a_X_1+a_ (X = Cl, Br; a = 0–0.5), have garnered significant attention.^[^ ^5^, ^6^ ^]^ However, a major drawback is their poor air stability: they degrade under humid conditions and generate H_2_S gas, primarily owing to the reaction between PS_4_ units and moisture.^[^ ^7^, ^8^, ^9^ ^]^ Although their high ionic conductivities (>1 mS cm^−1^) are partly attributed to the rotational motion of PS_4_^3−^ units, the impact of this dynamic behavior on their chemical stability remains unexplored.^[^ ^10^ ^]^ Several mitigation strategies have been proposed to address the issue of poor air stability. A widely explored approach involves incorporating absorbent materials into the SE matrix. For instance, Hayashi et al. reported that metal oxides such as Fe_2_O_3_, ZnO, and Bi_2_O_3_ can effectively capture the H_2_S generated during decomposition.^[^ ^11^, ^12^ ^]^ Similarly, Lee et al. embedded zeolites into Li_6_PS_5_Cl (LPSCl) to scavenge both H_2_S and H_2_O from humid air.^[^ ^13^ ^]^ Although these strategies effectively suppress toxic‐gas emissions, the electronically and ionically insulating nature of the additives compromises the ionic conductivity.

An alternative approach involves surface modification, wherein protective layers are created to minimize direct contact between the sulfide SE and humid air.^[^ ^14^, ^15^, ^16^, ^17^, ^18^, ^19^ ^]^ For example, Jung et al. demonstrated that a thin oxysulfide layer formed on LPSCl effectively suppressed surface degradation while preserving the integrity of the bulk structure.^[^ ^20^ ^]^ Kim et al. also developed a hydrophobic polydimethylsiloxane coating for LPSCl. The protected LPSCl retained 42.2% of its ionic conductivity, which decreased from 2.3 to 0.97 mS cm^−1^, after dry air exposure (at a dew point of −50 °C for 72 h), following a vacuum annealing treatment.^[^ ^21^ ^]^

Nevertheless, exploring moisture‐tolerant compositions is a fundamental strategy that can be integrated with the aforementioned approaches involving additives and surface modification. A widely adopted design principle is based on the hard and soft acid and base (HSAB) theory.^[^ ^22^, ^23^ ^]^ Replacing the hard acid P^5+^ with a softer acid such as Sn^4+^, As^5+^, or Sb^5+^ strengthens the bonding with sulfur, thereby reducing the reactivity of SEs with H_2_O.^[^ ^24^, ^25^, ^26^, ^27^, ^28^, ^29^, ^30^, ^31^, ^32^ ^]^ For instance, Kwak et al. developed Sb^5+^‐substituted phosphorus‐free Li_4‐_ *_x_* Sn_1‐_ *_x_* Sb*_x_* S_4_, which exhibited significantly enhanced air stability compared with that of LPSCl.^[^ ^33^ ^]^ Although such substitutions may modestly improve the ionic conductivity, they often involve trade‐offs such as increased material density, higher cost, and reduced reduction stability.^[^ ^34^ ^]^ Additionally, replacing the hard base sulfur with oxygen can reinforce the P–O bond, further improving moisture resistance.^[^ ^35^ ^]^ However, increasing the oxygen content typically reduces the ionic conductivity owing to the smaller ionic radius and higher electronegativity of O compared with S.^[^ ^36^ ^]^

Notably, Al^3+^ doping has been reported to simultaneously enhance the ionic conductivity and air stability of LPSCl.^[^ ^37^ ^]^ However, the preferred incorporation site of Al^3+^ remains controversial. Zhang et al. reported that Al^3+^ exclusively substitutes at the Li sites without occupying the P sites;^[^ ^38^ ^]^ however, another study proposed that Al^3+^ may also substitute for P, thereby contributing to improved chemical and electrochemical robustness.^[^ ^39^ ^]^ Meanwhile, theoretical calculations have suggested that Se substitution can reduce H_2_O adsorption in sulfide SEs;^[^ ^40^ ^]^ however, this effect has not been experimentally verified in argyrodite systems.

Although previous studies based on the HSAB concept have predominantly focused on soft‐acid substitution at the P site, substitution at the Li site remains largely unexplored. Given that Li‐site cations can also influence both the structural and chemical stability of sulfide SEs,^[^ ^41^ ^]^ incorporating a hard acid at the Li site in combination with a soft base at the S site may offer an alternative route to improve air stability while preserving high ionic conductivity.

Despite extensive efforts to improve air stability, the decomposition mechanisms of sulfide SEs under humid conditions remain incompletely understood, presenting a major challenge for further advancement. The currently accepted degradation pathway proceeds as follows. Initially, H_2_O molecules adsorb onto the surface of the argyrodite, representing a mild and reversible form of degradation that can be mitigated via vacuum annealing.^[^ ^8^, ^42^ ^]^ Prolonged exposure initiates reactions with the adsorbed moisture to form LiOH, LiCl, and their hydrates, resulting in irreversible decomposition. These LiOH‐related species react with atmospheric CO_2_ to form Li_2_CO_3_.^[^ ^43^ ^]^ Concurrently, the loss of Li and Cl from the Li_6‐_ *_y_* PS_5‐_ *_y_* X_1+_ *_y_* framework transforms PS_4_^3−^ units into P_2_S_6_^4−^ species, in conjunction with sulfur–oxygen exchange reactions that yield phosphate and sulfate byproducts.^[^ ^43^ ^]^ This humidity‐induced degradation results in morphological deterioration, structural collapse, and the accumulation of resistive side products, which ultimately impair the electrochemical performance of sulfide‐based ASSBs.^[^ ^44^ ^]^ However, as these degradation pathways have only recently been elucidated, studies investigating the H_2_O adsorption energies on argyrodite surfaces or the kinetics of subsequent decomposition processes remain limited and key aspects of air stability mechanisms continue to be largely unexplored.

Building on these insights, we introduce a newly engineered Li‐argyrodite electrolyte featuring an Al/Se co‐substituted composition, Li_6‐3_ *_x_* Al*_x_* PS_5‐1.5_ *_x_* Se_1.5_ *_x_* Cl. This strategy was devised based on the recognition that Li vacancies enhance ionic mobility, and that S‐site chemistry critically influences surface hydrolysis. Accordingly, Al was introduced at the Li sites and Se was introduced at the S sites to concurrently optimize the ionic conductivity and air stability. Structural analyses employing X‐ray diffraction (XRD) and Rietveld refinement validated the successful incorporation of Al and Se into the targeted lattice sites. The optimized composition, Li_5.850_Al_0.050_PS_4.925_Se_0.075_Cl (AS‐LPSCl, *x* = 0.05), exhibited a high Li^+^ conductivity of 4.91 mS cm^−1^ at 30 °C. Notably, AS‐LPSCl demonstrated substantially enhanced resistance to surface degradation under humid conditions (a dew point of −40 °C), outperforming pristine LPSCl. Theoretical calculations indicated less favorable H_2_O adsorption on AS‐LPSCl. Ab‐initio molecular dynamics (AIMD) calculations further revealed critical mechanistic insights: the Se substitution restricted the rotational freedom of the surface PS_4_^3−^ tetrahedra, thereby suppressing the exposure of new reactive sites to moisture. In parallel, electronic effects from Al and Se substitution reduce the thermodynamic driving force for H_2_O adsorption. Consistent with these findings, AS‐LPSCl exhibited a minor decrease in discharge capacity after air exposure in NCMǀSEǀ(Li‐In) half‐cells.

## 2 Results and Discussion

**Figure 1:**
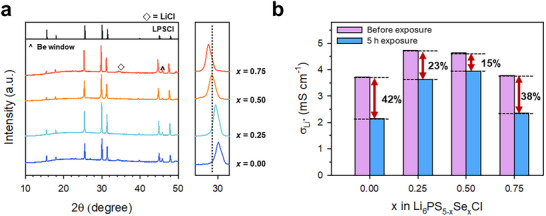
Characterization of Se‐substituted Li_6_PS_5_Cl, Li_6_PS_5‐_ *_x_* Se*_x_* Cl (*x* = 0.00, 0.25, 0.50, and 0.75). a) XRD patterns and b) Li^+^ conductivities before and after exposure to dry air with a dew point of −40 °C.

A series of Se‐substituted Li_6_PS_5‐_ *_x_* Se*_x_* Cl (*x* = 0.00, 0.25, 0.50, and 0.75) samples were synthesized via mechanochemical milling, followed by annealing at 550 °C under an Ar flow. The XRD patterns confirmed that all the samples retained the characteristic crystal structure of LPSCl ($F\bar{4}3m$: space group no. 216), with minor impurity peaks observed at *x* = 0.75 (**Figure** **1**a). A systematic shift of the (311) reflection toward lower angles at ≈30° indicated the successful incorporation of larger Se^2−^ ions (198 pm) in place of S^2−^ (184 pm) (Figure S1, Supporting Information). The Li^+^ conductivity was evaluated using ion‐blocking Ti|SE|Ti symmetric cells via AC impedance spectroscopy. The Se substitution led to a marginal improvement in the Li^+^ conductivity, which is attributed to an expansion of the Li⁺ diffusion pathways and increased anion polarizability.^[^ ^45^, ^46^ ^]^ The air stability was evaluated by storing powder samples in a controlled chamber maintained at a dew point of −40 °C (a relative humidity of 0.8–0.9% at room temperature; Figure S2, Supporting Information) for 5 h. The Li^+^ conductivities at 30 °C before and after exposure, in conjunction with their retention ratios, are summarized in Figure 1b. The conductivity of the pristine LPSCl decreased from 3.71 to 2.15 mS cm^−1^, corresponding to 58% retention. By contrast, the Se substitution significantly improved the air stability; Li_6_PS_4.5_Se_0.5_Cl retained 85% of its initial conductivity and exhibited substantially reduced H_2_S emissions (Figure 1b; Figure S3, Supporting Information). These findings are consistent with previous reports,^[^ ^40^ ^]^ indicating that the enhanced air stability resulted from weakened H_2_O adsorption and reduced surface activation on Se‐substituted compositions. However, the introduction of Se compromised the electrochemical stability. Cyclic voltammetry (CV) at 30 °C (Figure S4a, Supporting Information) revealed that Li_6_PS_4.5_Se_0.5_Cl exhibited higher oxidative currents than LPSCl, indicating reduced oxidative stability. Consequently, the Se‐substituted sample led to inferior rate performances in NCM||(Li‐In) half‐cells operated between 3.0 and 4.3 V (vs Li/Li^+^) when employed as a catholyte (Figure S4b, Supporting Information). These results underscore the need for further strategies to balance the trade‐off between enhanced air stability and electrochemical performance, guiding the exploration of multi‐element substitutions involving both Al and Se, specifically employing Al_2_Se_3_ incorporation.

**Figure 2:**
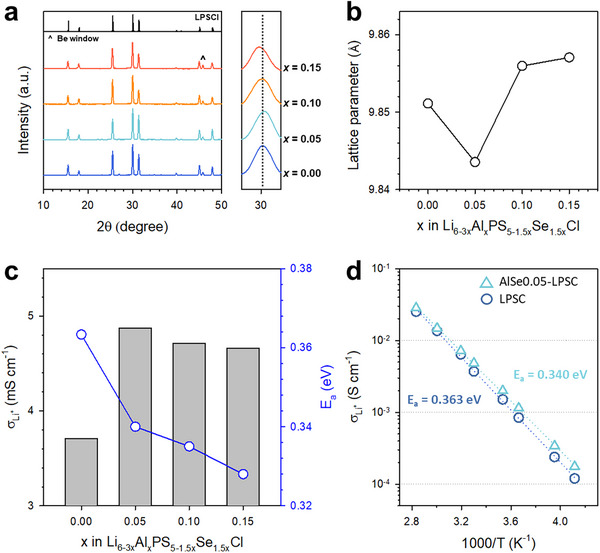
Characterization of Al/Se co‐substituted Li_6_PS_5_Cl, Li_6‐_ *_x_* Al*_x_* PS_5‐1.5_ *_x_* Se_1.5_ *_x_* Cl (*x* = 0–0.15). a) XRD patterns, b) lattice parameters, c) Li^+^ conductivity at 30 °C (left axis, bar graph), and activation energy (right axis, line graph) as functions of *x*. d) Arrhenius plots of Li^+^ conductivity for Li_5.850_Al_0.050_PS_4.925_Se_0.075_Cl, compared with Li_6_PS_5_Cl.

To investigate the extent of Al_2_Se_3_ incorporation into the LPSCl structure, Li_6‐3_ *_x_* Al*_x_* PS_5‐1.5_ *_x_* Se_1.5_ *_x_* Cl samples with *x* = 0.00−0.15 were synthesized. The XRD patterns confirmed the retention of the cubic $F\bar{4}3m$ phase across all compositions without any detectable impurity phases (**Figure** **2**a). Notably, the (311) diffraction peak near 30° shifted to a higher angle at *x* = 0.05, followed by a gradual shift back to lower angles at *x* = 0.10 and 0.15. This trend was corroborated by refined lattice parameters (Figure 2b): the lattice constant decreased from 9.8511 Å (*x* = 0.00) to 9.8436 Å (*x* = 0.05), and subsequently increased to 9.8560 Å and 9.8571 Å at *x* = 0.10 and 0.15, respectively. These observations suggest that Al^3+^ (39 pm) preferentially substituted for Li^+^ (59 pm), resulting in the initial lattice shrinkage. If Al^3+^ were to substitute for P^5+^ (17 pm), continuous lattice expansion would be expected owing to the larger radii of Al^3+^ and Se^2−^ relative to P^5+^ and S^2−^, respectively (Figure S1, Supporting Information). A similar lattice contraction trend was also observed in Li_6‐3_ *_x_* Al*_x_* PS_5_Cl samples, further supporting the preferential Li‐site occupancy of Al^3+^ (Figure S5, Supporting Information). Rietveld refinement results of synchrotron‐based high‐resolution powder diffraction of *x* = 0.05 are shown in Figure S6 and Table S1 (Supporting Information). These combined trends, together with the Rietveld refinement results, consistently confirm the preferential incorporation of Al^3+^ into the Li sites in the Al/Se co‐substituted argyrodites.

The Li^+^ conductivities and activation energies (E_a_) of Li_6‐3_ *_x_* Al*_x_* PS_5‐1.5_ *_x_* Se_1.5_ *_x_* Cl are summarized in Figure 2c, and the corresponding Arrhenius plots for pristine (*x* = 0.00) and substituted (*x* = 0.05) compositions are shown in Figure 2d. The Meyer–Neldel energies (Δ_0_) of Li_6‐3_ *_x_* Al*_x_* PS_5‐1.5_ *_x_* Se_1.5_ *_x_* Cl was 32 meV, indicating that a decrease in E_a_ leads to an enhancement in ionic conductivity (Table S2 and Note S1, Supporting Information). Even a small degree of substitution at *x* = 0.05 resulted in a notable increase in Li^+^ conductivity from 3.71 to 4.91 mS cm^−1^. Further increases in the Al content at *x* ≥0.05 consistently yielded conductivities exceeding 4.5 mS cm^−1^. Concurrently, the activation energy progressively decreased from 0.363 to 0.328 eV, which indicated that the Al and Se co‐substitution facilitated Li⁺ transport.

**Figure 3:**
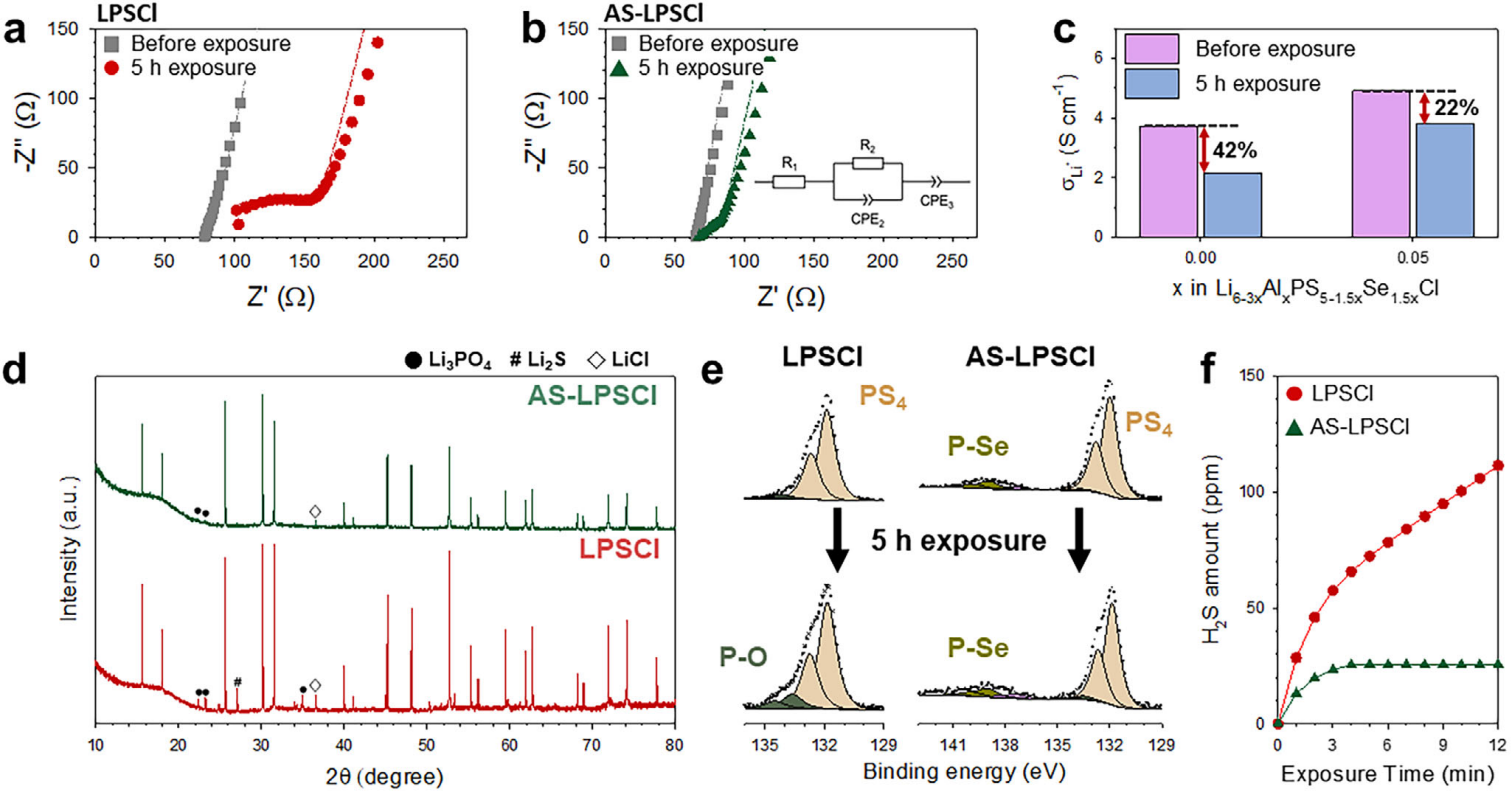
Air stability of Al/Se co‐substituted Li_5.850_Al_0.050_PS_4.925_Se_0.075_Cl (AS‐LPSCl) compared with pristine LPSCl. Nyquist plots of SEs before and after 5 h of dry‐air exposure (at a dew point of −40 °C) for a) pristine LPSCl and b) AS‐LPSCl, with an equivalent circuit used for the fitting shown in the inset of (b). c) Li^+^ conductivities before and after exposure. d) Synchrotron HRPD patterns after exposure. e) P 2p XPS spectra before and after exposure. f) Quantified H_2_S evolution over time during exposure.

The air stability of the Al_2_Se_3_‐substituted LPSCl was evaluated under the conditions described previously (5 h exposure to dry air at a dew point of −40 °C). Among the tested compositions, AS‐LPSCl (Li_5.850_Al_0.050_PS_4.925_Se_0.075_Cl) was selected for further evaluation because it exhibited the highest Li^+^ conductivity. Nyquist plots before and after exposure to pristine LPSCl and AS‐LPSCl are presented in **Figures** **3**a,b, respectively. Although the bulk resistance, which was associated with Li⁺ transport within individual particles, remained largely unchanged, the grain boundary resistance increased after exposure. This reflected surface degradation, which impeded Li⁺ migration across particles. The corresponding Li^+^ conductivities and their retentions after 5 and 72 h of exposure are summarized in Figure 3c and Table S3 (Supporting Information). Although the Al‐only substituted compositions (Li_6‐3_ *_x_* Al*_x_* PS_5_Cl) also exhibited improved air stability relative to LPSCl, their performances were inferior to those of AS‐LPSCl under identical conditions (Figures S7 and S8, Supporting Information), verifying the effectiveness of the co‐substitution approach.

Synchrotron‐based high‐resolution powder diffraction (HRPD) and X‐ray photoelectron spectroscopy (XPS) were employed to investigate the formation of byproducts following exposure to air, and the results are presented in Figures 3d and e. HRPD analysis revealed a lower intensity of impurities such as Li_3_PO_4_ and Li_2_S in AS‐LPSCl after exposure (Figure 3d). Consistently, the XPS results indicated that the Se substitution suppressed the hydrolysis of the PS_4_^3−^ units (Figure 3e). The predominant degradation pathway during air exposure involved the hydrolysis of P─S bonds within the PS_4_^3−^ tetrahedra in the argyrodite structure, where the ─OH groups from H_2_O attacked the P─S bonds, resulting in the formation of P─O bonds. This reaction led to the generation of oxy‐thiophosphate (P[S_4‐_ *_x_* O*_x_*]^3−^) or phosphate (PO_4_^3−^) species, which ultimately reduced the Li⁺ conductivity.^[^ ^47^ ^]^ In contrast to the distinct P–O signal near 133.5 eV observed in LPSCl, AS‐LPSCl exhibited a significantly lower P─O contribution of 5.1%, compared with 14.0% for LPSCl (Figure 3e; Table S4, Supporting Information), which indicated the effective suppression of moisture‐induced degradation by the Al and Se substitution. Furthermore, AS‐LPSCl released less than half the amount of H_2_S gas compared with that released by pristine LPSCl upon exposure (Figure 3f), further confirming its enhanced resistance to air‐induced decomposition.

According to a recently proposed mechanism for the surface degradation of LPSCl under dry‐room conditions, where only trace amounts of H_2_O are present but still sufficient to trigger reactions,^[^ ^48^ ^]^ the process proceeds via four key steps: i) H_2_O adsorption on the LPSCl surface; ii) substitution of surface S atoms by O; iii) rotation of P[S_4‐_ *_x_* O*_x_*]^3−^ tetrahedra, leading to the formation of an O‐rich surface; and iv) phase separation into smaller decomposition products, resulting in a porous surface. Among these steps, we focused on the initial H_2_O adsorption and polyhedral rotation of P[S_4‐_ *_x_* O*_x_*]^3−^ because they lead to surface inhomogeneity and continuous oxygen incorporation. To elucidate the effect of Al and Se co‐substitution on the air stability, a computational analysis was conducted to compare the adsorption affinity of H_2_O molecules and extent of P[S_4‐_ *_x_* O*_x_*]^3−^ tetrahedral rotations between LPSCl and AS‐LPSCl using (001) slabs constructed from each bulk structure (Figure S9 and Table S5, Supporting Information).

To identify variations in reactivity, two reaction equations were proposed: one for the H_2_O adsorption energy (*E*_ads_; Equation 1) and another for the S─O substitution energy (*E*_substitution_; Equation 2):

(1) $$E_{\mathrm{ads}}=E_{\mathrm{initial}+H_{2}O}-\;E_{\mathrm{initial}}-\;E_{H_{2}O}\;\left(\mathrm{eV}\right)$$

(2) $$E_{\mathrm{substitution}}=\frac{E_{\mathrm{substituted}}-\;E_{\mathrm{initial}}-n\left(\;\mu_{O}-\mu_{S}\right)}{n}\;\left(\mathrm{eV}/O\;\mathrm{atom}\right)$$

where *E*_initial_ and *E*_substituted_ denote the total energies of the structure before and after O substitution, respectively; µ_O_ and µ_S_ represent the chemical potentials of oxygen and sulfur, respectively; and *n* is the number of substituted O atoms. These expressions quantify the relative energetics of the H_2_O interactions and subsequent S‐to‐O exchange at the LPSCl surface.

Equation 1 was used to compare the adsorption favorability of AS‐LPSCl with that of pristine LPSCl. Prior to this comparison, the most stable H_2_O adsorption site on the surface of AS‐LPSCl was identified. Based on adsorption calculations performed for all plausible sites (Figure S10, Supporting Information), three candidates were found, as shown in Figure S11 (Supporting Information): i) adjacent to the Al site, ii) adjacent to the Se site, and iii) independent of either dopant. Among these, adsorption near the Al site exhibited the strongest energetic preference (–1.198 vs –0.757 vs –0.786 eV). Accordingly, the near‐Al site adsorption configuration, which exhibited the lowest E_ads_ value, was selected for evaluating the adsorption stability. The stronger adsorption at the Al‐adjacent site can be attributed to the S near Al having more dangling bonds. Because aliovalent Al doping reduces the local coordination number of S relative to non‐bonding S atoms in the bulk framework, the resulting under‐coordinated S atoms provide more favorable binding environments for H_2_O (Figure S12, Supporting Information). For comparison, the adsorption stability on the pristine LPSCl surface was also evaluated using the same methodology. As previously reported,^[^ ^48^ ^]^ H_2_O adsorption on LPSCl differs depending on whether H binds to a non‐bonding S atom (Wyckoff 4d site) or to a P─S bonding S atom (Wyckoff 16e site). As shown in Figure S13 (Supporting Information), the 16e site‐dependent adsorption yields the lower E_ads_ values, and therefore the most stable configuration (Figure S13g, Supporting Information) was selected for comparison with AS‐LPSCl.

**Figure 4:**
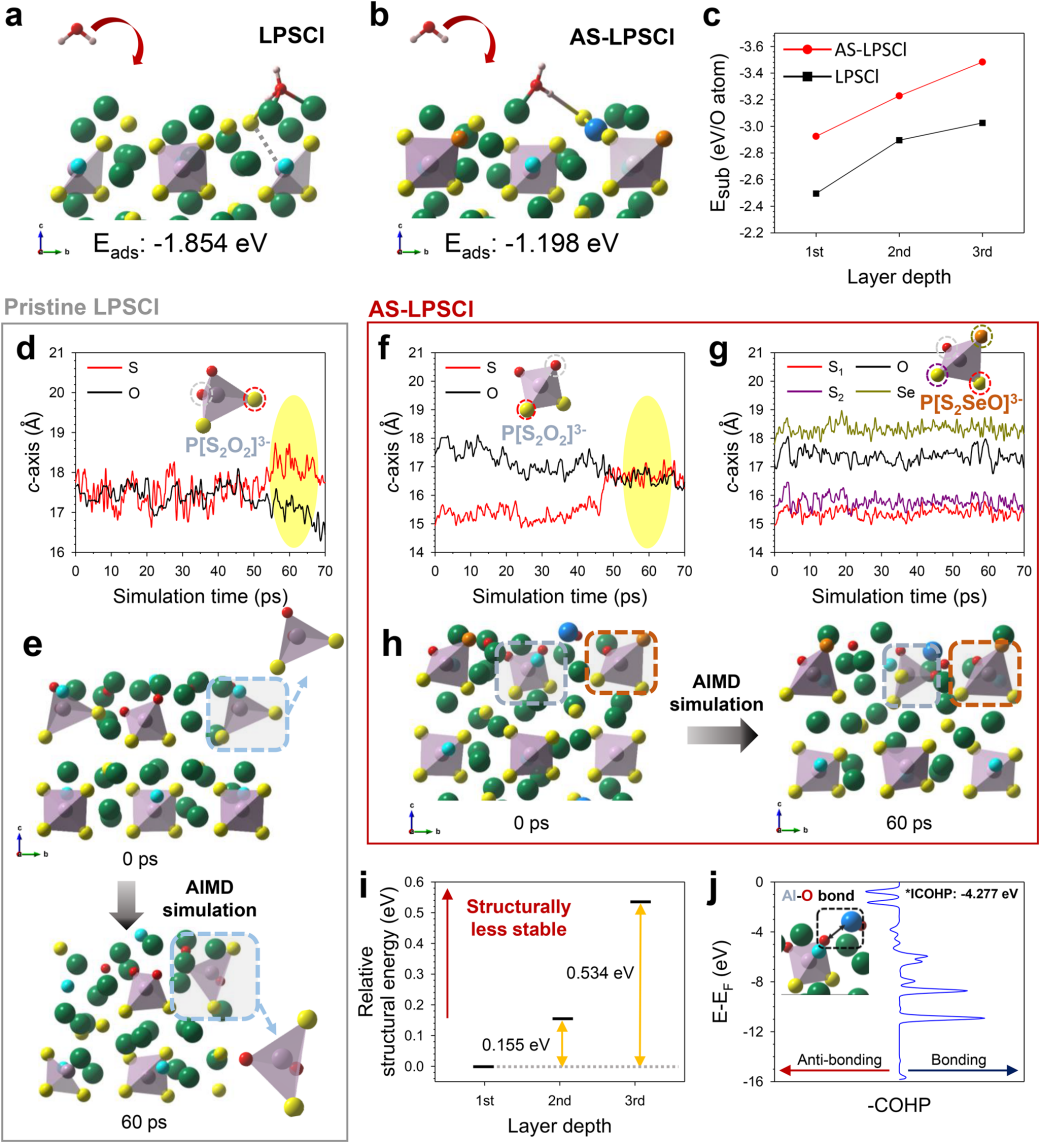
Computational comparison of air stability between AS‐LPSCl and LPSCl. E_ads_ calculations for a) LPSCl and b) AS‐LPSCl (Li: green, P: purple, S: yellow, Cl: cyan, Al: blue, Se: orange, O: red, and H: pink). c) Comparison of *E_substitution_* values of two mechanisms, additional substitution, and penetration. (d,f,g) Atomic displacement plots along the c‐axis and (e,h) AIMD simulation results for LPSCl (d, e) and AS‐LPSCl (f–h). i) Relative thermodynamic stability of Se layer‐substituted structures depending on the layer depth in comparison with the 1st layer‐substituted case. j) Plot of crystal orbital Hamiltonian populations (COHPs) for Al─O bond.

As shown in **Figures** **4**a,b, the less negative E_ads_ of AS‐LPSCl (–1.198 vs –1.854 eV for LPSCl) indicated less favorable H_2_O adsorption, thereby reducing surface reactivity toward moisture. Aliovalent Al substitution generated a Li‐deficient surface, which reduced the number of available adsorption sites. This theoretical hypothesis is supported by calculations on Al‐doped LPSCl (Al‐LPSCl) (Figure S14 and Table S5, Supporting Information), which show that a reduced surface Li concentration results in less stable H_2_O adsorption compared with pristine LPSCl (Figure S15, Supporting Information). In parallel, because of its lower electronegativity compared to S, Se could not effectively withdraw electrons from Li, resulting in a higher electron density around Li and weaker electrostatic interactions between Li and the oxygen of adsorbed H_2_O. Consequently, Se incorporation further suppressed the moisture‐induced reaction, which is consistent with previous reports.^[^ ^40^ ^]^

Furthermore, thermodynamic and kinetic approaches were employed to assess the degree of tetrahedral rotation. During successive O substitutions on the uppermost surface, further substitution competed with the penetration of O atoms into the deeper layers. For AS‐LPSCl, the reactions at the Se sites were excluded because the number of P─Se bonds remained unchanged upon exposure (Table S4, Supporting Information). As shown in Figure S16 (Supporting Information), all of the surface‐layer substitutions, including those at the 1st layer, 2nd layer, and combined sites, were thermodynamically more favorable than single‐atom penetration. Because the 1st and 2nd layers were directly exposed to the external environment (Figure S9c,f, Supporting Information), penetration was unfavorable until complete O substitution occurred in these surface layers of both materials. However, as shown in Figure 4c, the substitution in the 3rd layer became energetically more favorable than that in the 1st or 2nd layer, indicating a thermodynamic preference for downward migration regardless of the existence of dopants. Thus, it is anticipated that the penetration of oxygen atoms will take place after sufficient involvement of their amounts on the surface.

In AS‐LPSCl, the energy gap between the 1st‐ and 3rd‐layer substitutions was larger than that in LPSCl (–0.559 vs –0.530 eV per atom of O), suggesting a stronger thermodynamic driving force for O penetration. To assess whether this behavior was kinetically consistent, AIMD simulations were performed to track the rotational motion. In LPSCl, the S atoms initially occupied positions along the *c*‐axis (perpendicular to the surface), comparable to the O atoms, and gradually migrated toward the surface over time (Figures 4d,e). By contrast, AS‐LPSCl exhibited two distinct behaviors (Figure 4f−h): the P[S_2_O_2_]^3−^ tetrahedra required a longer time to align with O, whereas the P[S_2_SeO]^3−^ tetrahedra showed no rotational motion. This contrast became more evident with further substitution at the S‐exposed sites generated by prior rotations (Figure S17, Supporting Information).

This suppressed rotation in AS‐LPSCI can be attributed to the combined effects of the anion size and formation of supplementary Al─O bonds. As shown in Figure 4i, the Se substitution in deeper layers was substantially less stable than that on the outermost surface because of the structural stress imposed by the larger ionic radius of Se^2−^ (198 pm) compared with that of S^2−^ (184 pm). At the surface, Se anions could partially relieve this stress by shifting toward vacuum, whereas deeper layers were less capable of such relaxation. Thus, Se anions would prefer to remain at the top surface rather than migrate into the internal atomic layer through tetrahedral rotations. Furthermore, crystal orbital Hamiltonian population (COHP) analysis, which included the integrated COHP (ICOHP) value, confirmed the formation of Al─O bonds, with strongly negative values reflecting a high binding affinity (Figure 4j). This robust bonding network restricted vibrational motion, thereby serving as a secondary factor that suppressed tetrahedral rotations.

**Figure 5:**
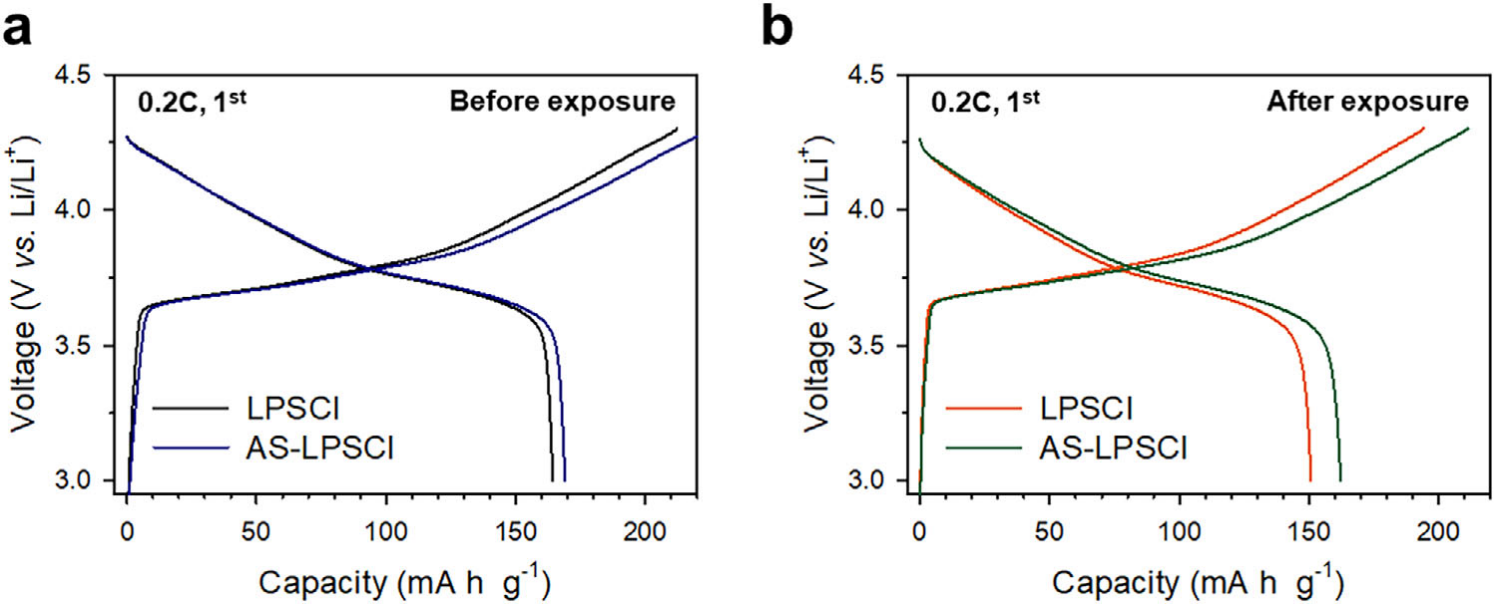
Electrochemical performances of NCM electrodes employing LPSCl and AS‐LPSCl before and after air exposure in NCM|SE|Li‐In cells at 0.2C and 30 °C. Initial charge–discharge profiles for employing LPSCl and AS‐LPSCl a) before air exposure and b) after 5 h of air exposure (at a dew point of −40 °C).

Finally, the electrochemical performance of AS‐LPSCl was evaluated in NCMǀLPSClǀ(Li–In) cells in a range of 3.0–4.3 V (vs Li/Li^+^) at 30 °C, as shown in **Figure** **5**. The initial cycling was conducted at 0.2C, followed by rate capability tests at 0.5C, 1C, and 2C. At 0.2C, the AS‐LPSCl electrodes delivered a slightly higher discharge capacity than pristine LPSCl (Figure 5a), which is attributed to its enhanced Li^+^ conductivity. The charge–discharge voltage profiles of the cells assembled with air‐exposed SEs are shown in Figure 5b. Although the electrodes exhibited comparable initial capacities prior to exposure, the AS‐LPSCl electrode demonstrated substantially improved capacity retention upon exposure to air. Specifically, the LPSCl electrodes exhibited a 14 mAh g^−1^ decrease in discharge capacity (from 164 to 150 mAh g^−1^), whereas the AS‐LPSCl electrodes exhibited a smaller decrease of only 7 mAh g^−1^ (from 169 to 162 mAh g^−1^). The rate performance results are presented in Figure S18 and Table S6 (Supporting Information) summarizes the capacity retention values across all C‐rates before and after exposure. These results confirmed that AS‐LPSCl consistently outperformed pristine LPSCl in maintaining its electrochemical performance following air exposure.

## 3 Conclusion

In this study, we developed an Al and Se co‐substituted argyrodite, Li_6‐3_ *_x_* Al*_x_* PS_5‐1.5_ *_x_* Se_1.5_ *_x_* Cl, which exhibited an improved Li⁺ conductivity of 4.91 mS cm^−1^ at 30 °C (*x* = 0.05, compared with 3.71 mS cm^−1^ for the pristine sample) and a superior retention of 85% (compared with 58%) upon dry‐air exposure (at a dew point of −40 °C). This co‐substitution strategy effectively addressed the compromised electrochemical stability observed in the Al‐only substituted analogs. Complementary analyses employing HRPD and XPS confirmed the reduced impurity formation and significantly lower surface oxidation. The theoretical analyses consistently indicated less favorable H_2_O adsorption on AS‐LPSCl compared with LPSCl. Moreover, AIMD calculations revealed that the incorporation of Al and Se into the lattice inhibited the rotation of P[S_2_O_2_]^3−;^ and P[S_2_SeO]^3−^ tetrahedra, which otherwise exposes new S sites to moisture. Preferential surface‐oriented Se^2−^ and Al─O interactions suppressed this degradation pathway by minimizing direct H_2_O interactions. The enhanced moisture tolerance of AS‐LPSCl over pristine LPSCl was further validated by the improved capacity retention in NCMǀLPSClǀ(Li–In) half‐cells following air exposure. Collectively, these findings underscore the polyhedral rotation‐driven moisture affinity as an overlooked degradation mechanism and establish Al and Se co‐substitution as a promising strategy for simultaneously enhancing ionic transport and air stability in sulfide‐based SEs. Extending this design principle to other SE systems and exploring diverse substitution chemistries may further advance the development of environmentally robust high‐performance ASSBs.

## 4 Experimental Section

### Preparation of Materials

Li_6_PS_5_Cl (LPSCl) powder was synthesized via ball milling and subsequent annealing under an Ar atmosphere. After a stoichiometric mixture of Li_2_S (99.9%, Alfa Aesar), P_2_S_5_ (99%, Sigma–Aldrich), and LiCl (99.99%, Sigma–Aldrich) were ball‐milled at 600 rpm for 3 h in a ZrO_2_ vial with ZrO_2_ balls using Pulverisette 7PL (Fritsch GmbH), heat treatment (HT) was conducted at 550 °C for 12 h under an Ar atmosphere. Al‐LPSCl powder was synthesized using a stoichiometric mixture of Li_2_S, P_2_S_5_, LiCl, and Al_2_S_3_ (99%, Alfa Aesar). Se‐LPSCl powder was synthesized using a stoichiometric mixture of Li_2_S, P_2_S_5_, LiCl, P (99.99%, Sigma–Aldrich), and Se (99.999%, Sigma–Aldrich). Al_2_Se_3_‐LPSCl (AS‐LPSCl) powder was synthesized using a stoichiometric mixture of Li_2_S, P_2_S_5_, LiCl, and Al_2_Se_3_ (99%, Sigma–Aldrich).

### Material Characterizations

Powder XRD patterns were collected utilizing a Rigaku MiniFlex600 diffractometer with Cu Kα radiation (*λ* = 1.5406 Å). XRD cells containing hermetically sealed SE samples were analyzed using a Be window mounted on an XRD diffractometer at 40 kV and 15 mA. HRPD patterns were acquired at the Pohang Accelerator Laboratory (PAL) 9B HRPD beamline using a Si (111) double‐crystal monochromator (DCM) with a monochromatic X‐ray wavelength of 1.546 Å (LPSCl and AS‐LPSCl). The Rietveld refinement method was employed to refine the diffraction data using the Fullprof software. The samples were mounted on a sample holder in an Ar‐filled glove box and transferred to the XPS instrument without air exposure. SEM images and corresponding EDXS elemental maps were obtained using a Crossbeam 540 (Zeiss). The sample specimens were stored and transported using an air‐isolation system holder to prevent exposure to ambient air. To investigate the air stability under dry room conditions with a dew point of −40 °C, 200 mg powder samples (placed on a vial cover) were placed in a custom‐made container for a fixed duration. After exposure, the samples were transferred to an Ar‐filled glove box using an airtight desiccator. The dew point was controlled within an acceptable error range of ± 2 °C using a cooling dehumidifier (Figure S2, Supporting Information). The ionic conductivities of the air‐exposed samples were measured in the absence of HT.

### Electrochemical Characterizations

The Li^+^ conductivity was measured via the AC impedance method using Li^+^‐blocking Ti|SE|Ti symmetric cells (Φ = 6 mm). Cold‐pressed pellets were prepared at an applied pressure of 370 MPa. The EIS data were recorded at an amplitude of 100 mV and a frequency range of 10 to 7 MHz using a VMP3 (Bio‐Logic). Li‐In counter and reference electrodes were used for the CV measurements. Li‐In counter electrodes, comprising a partially lithiated indium composition (Li_0.5_In), were synthesized by mixing lithium powder (FMC Lithium Corp.) with indium powder (Sigma–Aldrich, 99%). The LPSCl powder (150 mg) was pelletized at 70 MPa to form the SE layers. Composite working electrodes were prepared from a mixture of SE and Super C65 powders in a weight ratio of 70:30:3. The working and Li‐In counter electrodes were then placed on each side of the SE layer. Finally, the assemblies were pressed at 370 MPa and room temperature (RT). The CV cells were tested under an external pressure of 70 MPa at 30 °C. The scan rate was 0.1 Mv s^−1^. The mass loadings of the working and counter electrodes of the cells used in the CV experiments were 11.2 and 45.1 mg cm^−2^, respectively. Li‐In counter and reference electrodes were used for all‐solid‐state half‐cells. LPSCl powder (150 mg) was pelletized at 70 MPa to form the SE layers. A LiNbO_3_‐coated LiNi_0.7_Co_0.15_Mn_0.15_O_2_ (NCM711) powder was used in this study. The composite working electrodes were prepared from a mixture of NCM, SE, and Super C65 powders in a weight ratio of 70:30:3. Subsequently, the working (7.66 mg cm^−2^) and Li‐In (45.1 mg cm^−2^) counter electrodes were placed on each side of the SE layers. Finally, the assemblies were pressed at 370 MPa and RT. The all‐solid‐state cells were assessed under an external pressure of 70 MPa at 30 °C.

### Theoretical Calculations

DFT calculations were performed employing the generalized gradient approximation (GGA) with the Perdew–Burke–Ernzerhof (PBE) functional in the Vienna Ab initio Simulation Package (VASP).^[^ ^49^, ^50^ ^]^ The Al and Se‐doped and Al single‐doped argyrodite structures were prepared utilizing the Supercell program,^[^ ^51^ ^]^ which provided the top 30 electrostatically stable structures based on the Li_6_PS_5_Cl structure from the Materials Project database.^[^ ^52^ ^]^ Slab structures for the (001) and (010) surfaces, constructed from these bulk structures and possessing sufficient vacuum (≥15 Å) along the [001] and [010] directions, were applied in the surface‐related calculations. The energy cutoff of the plane‐wave basis set was 520 eV. Ionic positions were fully relaxed until the interatomic force was less than 0.02 eV Å^−1^ for bulk and slab structures. For Brillouin zone sampling, the k‐point meshes were set to 5 × 5 × 5 for the bulk structures and 3 × 3 × 1 for the slab structures. Van der Waals interactions were considered in the water‐adsorption calculations employing the DFT‐D3 method with the Becke–Johnson damping function.^[^ ^53^ ^]^ ICOHP calculations and COHP plotting were performed using the local orbital basis suite toward electronic‐structure reconstruction (LOBSTER) software and Pymatgen library.^[^ ^54^, ^55^, ^56^, ^57^, ^58^ ^]^ AIMD simulations were performed with a 1 × 1 × 1 k‐point grid and the NVT ensemble simulated using a Nose–Hoover thermostat.^[^ ^59^ ^]^ Post‐analyses of the atomic displacement along the *c*‐axis were conducted using the Pymatgen library and smoothed using a Savitzky–Golay filter.

## Conflict of Interest

The authors declare no conflict of interest.

## Supporting information



Supporting Information

## Data Availability

The data that support the findings of this study are available from the corresponding author upon reasonable request.
