# Supplementary material for: Improved Air Stability of Li Argyrodites Through PS4 3− Rotation Suppression by Al and Se Co‐Substitution for All‐Solid‐State Batteries
Source: Adv Sci (Weinh). 2025 Dec 16;13(11):e19093. doi: 10.1002/advs.202519093 (PMC12931221; doi:10.1002/advs.202519093)
Supplement: Supplementary file 1 — Supporting Information [file ADVS-13-e19093-s001.docx]

**Improved Air Stability of Li Argyrodites through** **PS_4_^3-^ Rotation Suppression by Al and Se Co-substitution for All-Solid-State Batteries**

*Juhyoun Park*^†^*, Jihun Lee*^†^*, Yoon-Seong Kim*^†^*, Donghyeok Kim, Minseo Jang, Junwoo Lee, Hae-Yong Kim, Changhun Park, Jeongheon Kim, Habin Chung, Kyung-Wan Nam, Dong-Hwa Seo*, and Yoon Seok Jung**

J. Park, J. Lee, D. Kim, M. Jang, Prof. Y. S. Jung
Department of Chemical and Biomolecular Engineering, Yonsei University, Seoul 03722, Republic of Korea

E-mail: [yoonsjung@yonsei.ac.kr](mailto:yoonsjung@yonsei.ac.kr)

Prof. Y. S. Jung
Department of Battery Engineering, Yonsei University, Seoul 03722, Republic of Korea

Y.-S. Kim, Prof. D.-H. Seo
Department of Materials Science and Engineering, Korea Advanced Institute of Science and Technology (KAIST), Daejeon 34141, Republic of Korea
E-mail: [dseo@kaist.ac.kr](mailto:dseo@kaist.ac.kr)

C. Park, J. Kim, H. Chung
LG Chem, Ltd. LG Science Park, 30, Magokjungang 10-ro, Gangseo-gu, Seoul 07796, Republic of Korea

H.-Y. Kim, K.-W. Nam

Department of Energy and Materials Engineering, Dongguk University, 30 Pildong-ro 1-gil, Jung-gu, Seoul 04620, Republic of Korea

[^†^] These authors contributed equally to this work.

**Table of Contents**

1. Supporting Figures
2. Supporting Tables

1. Supporting Figures


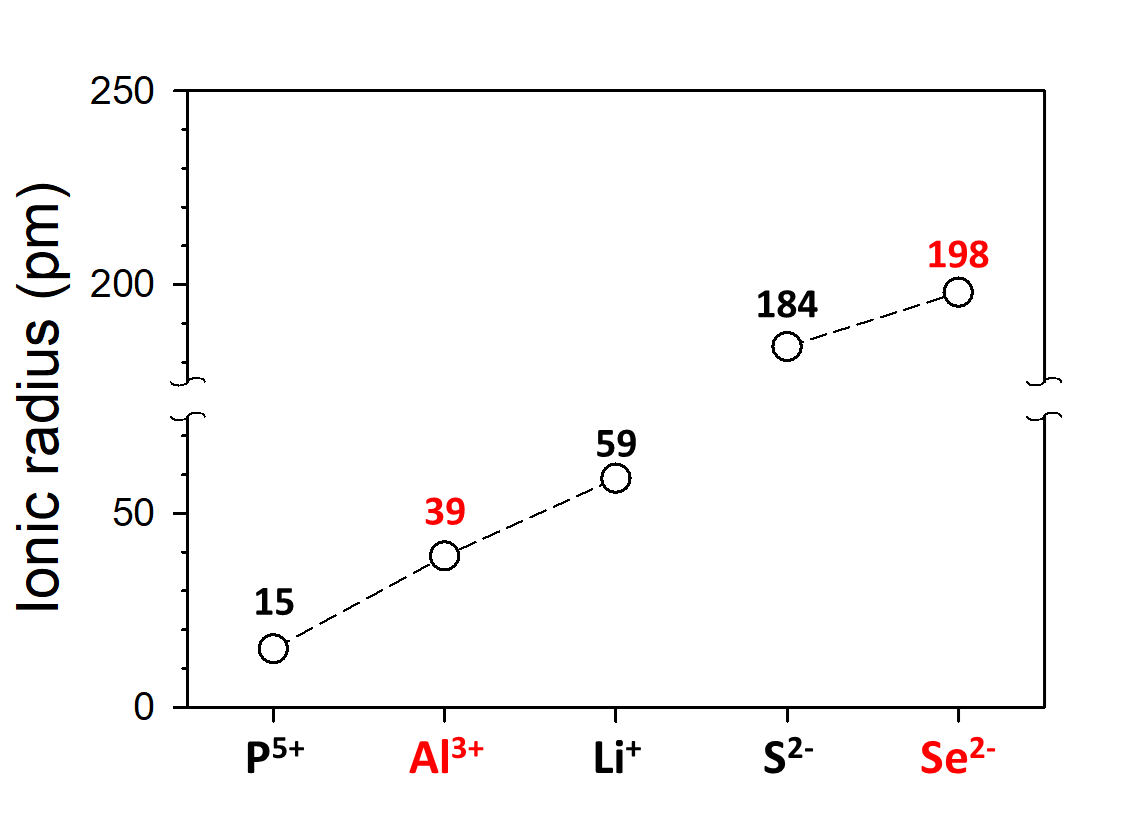


**Figure S1.** Ionic radii of P^5+^, Al^3+^, and Li^+^ in coordination number 4, and those of S^2-^ and Se^2-^ in coordination number 6.


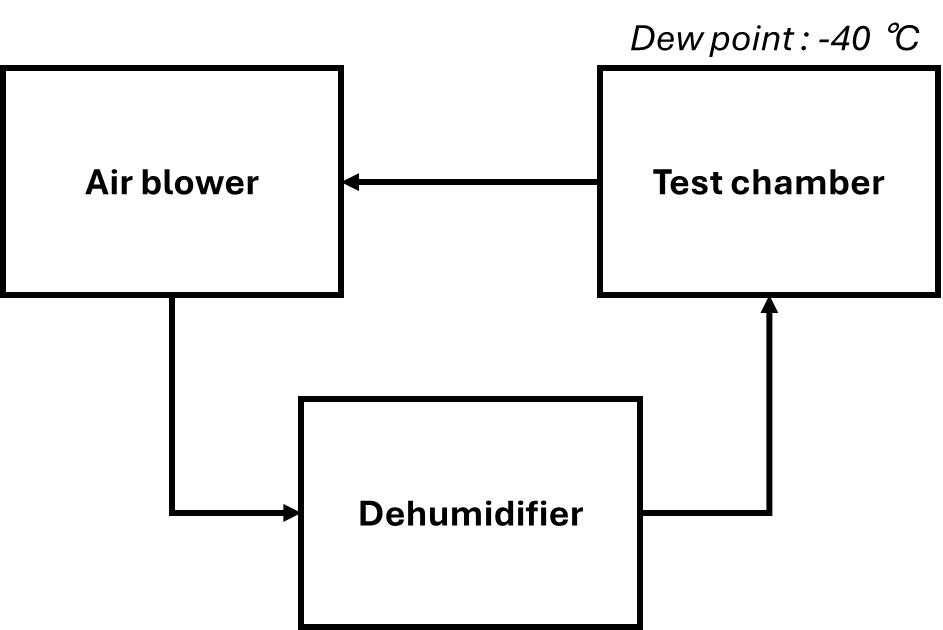


**Figure S2**. Schematic of the test chamber used for air-stability evaluation.


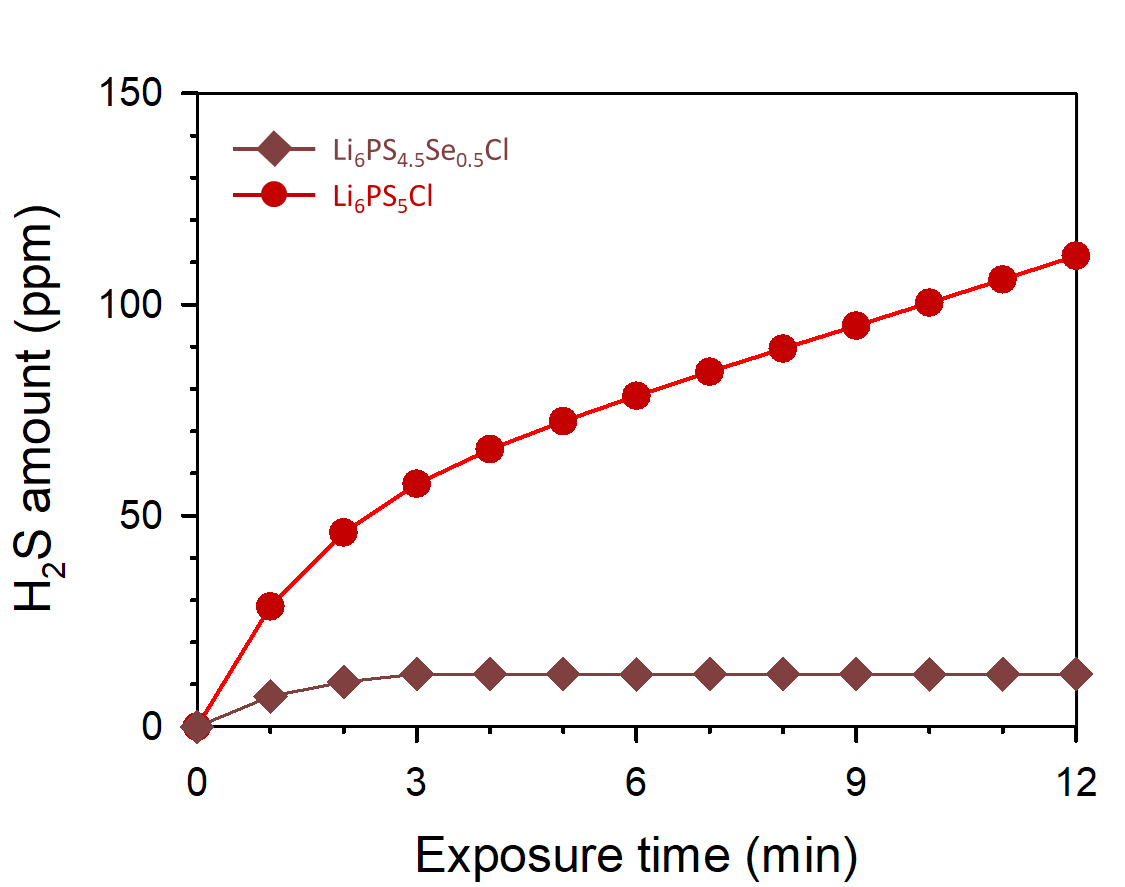


**Figure S3**. H_2_S evolution as a function of time in dry air with a dew point of -40 °C for Li_6_PS_4.5_Se_0.5_Cl compared to that of Li_6_PS_5_Cl.


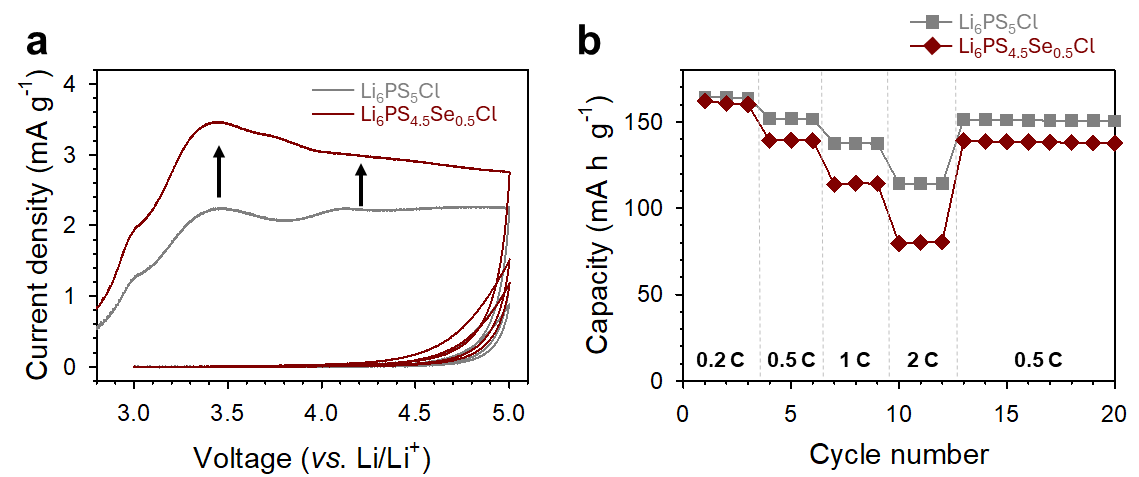


**Figure S4.** Electrochemical stability of Li_6_PS_4.5_Se_0.5_Cl compared with that of Li_6_PS_5_Cl at 30 °C. a) Results of cyclic voltammetry in (SE-C)ǀLi_6_PS_5_Clǀ(Li-In) cells at 0.1 mV s^-1^. b) Rate capabilities in NCMǀLi_6_PS_5_Clǀ(Li-In) cells.


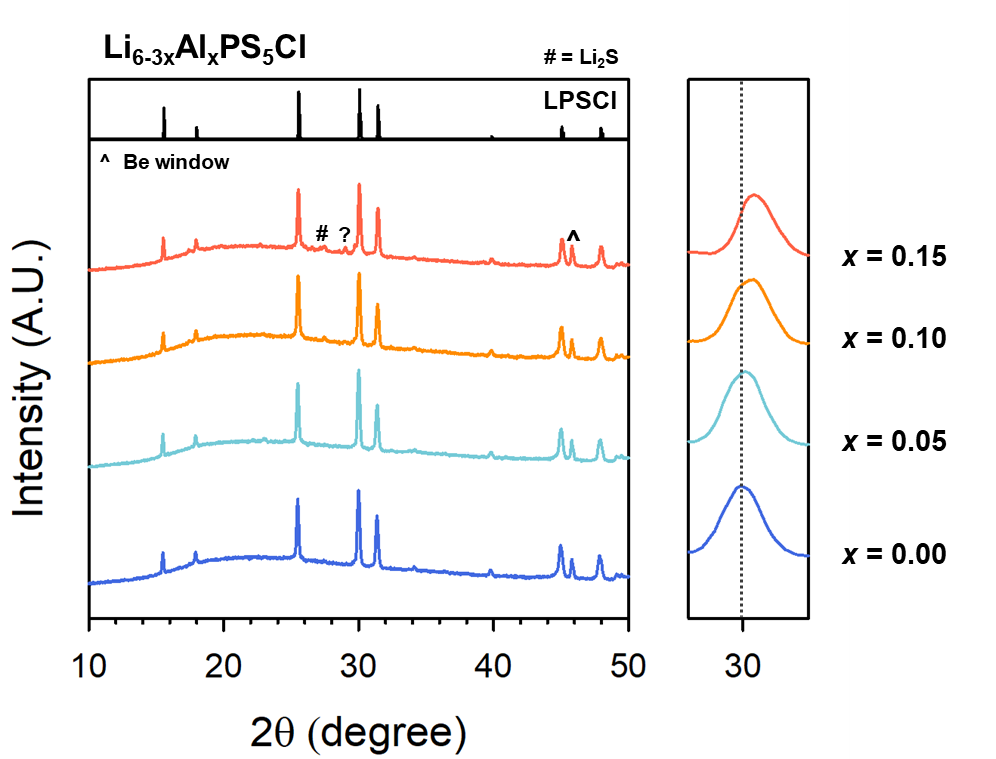


**Figure S5.** XRD patterns of Li_6-3_*_x_*Al*_x_*PS_5_Cl (*x* = 0.00–0.15) showing lattice shrinkage of the cubic argyrodite phase upon Al substitution.


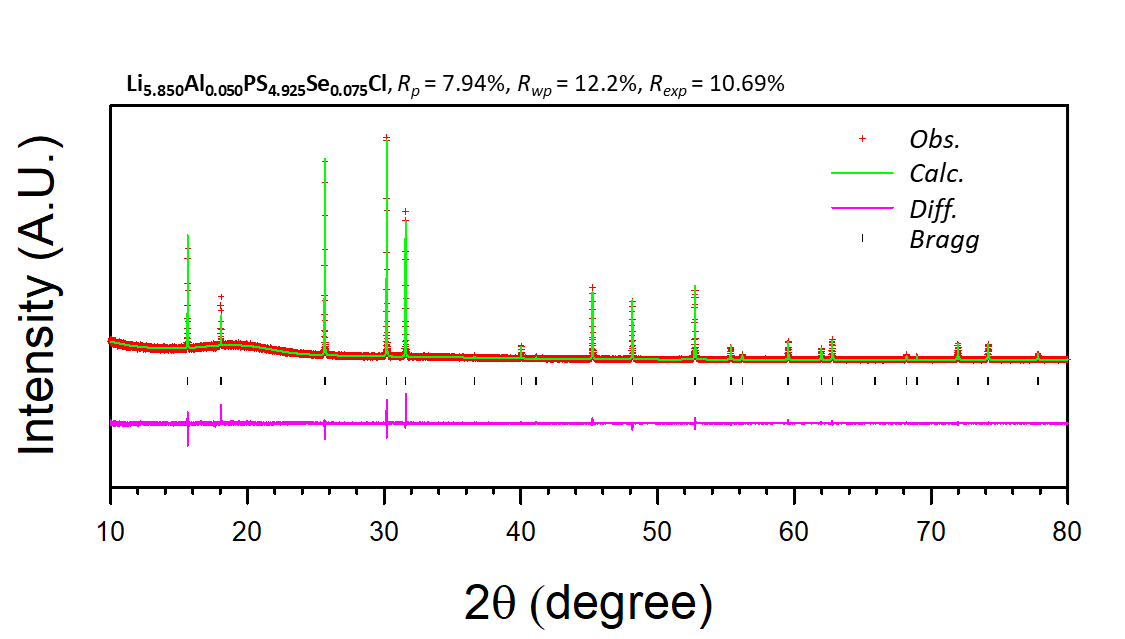


**Figure S6.** HRPD Rietveld refinement results. Observed and calculated HRPD Rietveld refinement profiles of Li_5.850_Al_0.050_PS_4.925_Se_0.075_Cl.


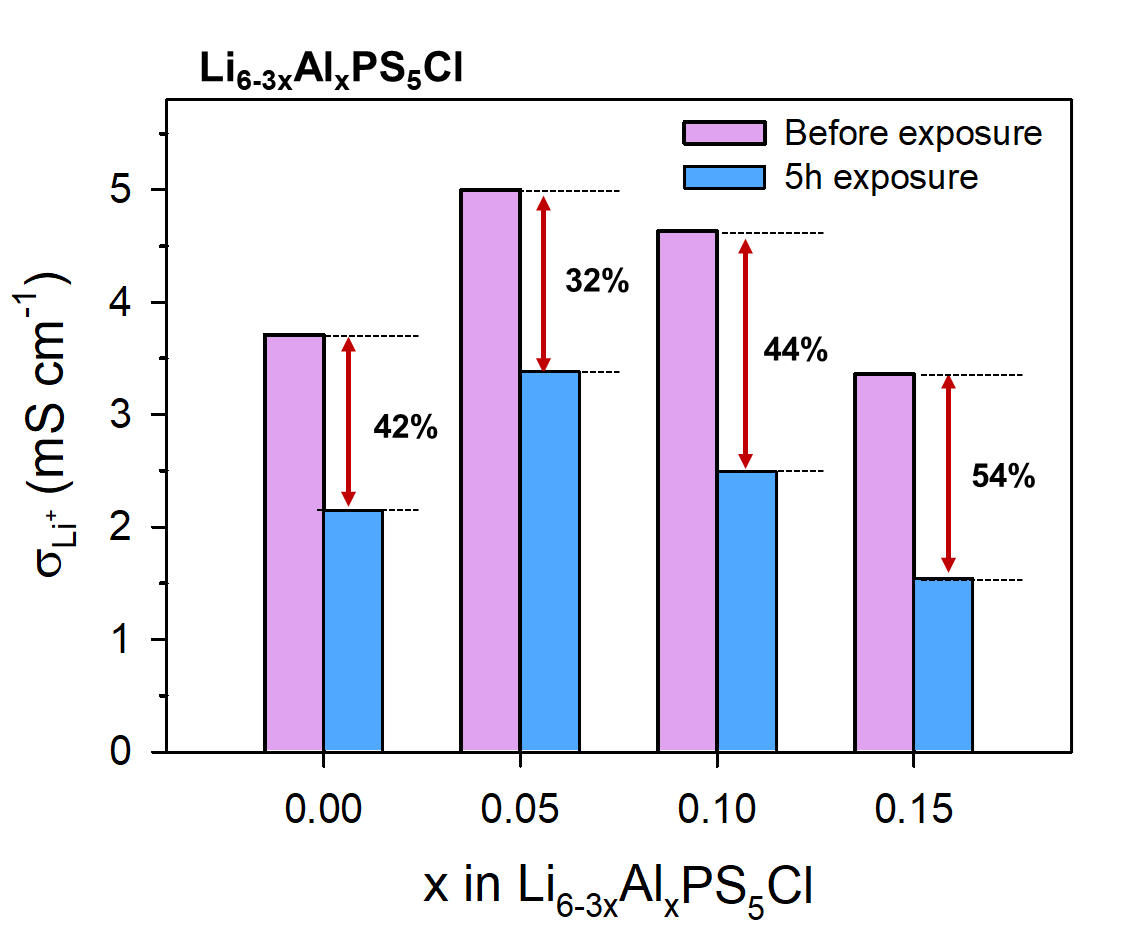


**Figure S7.** Li^+^ conductivities of Li_6-3_*_x_*Al*_x_*PS_5_Cl before and after air exposure (at a dew point of −40 °C for 5 h).


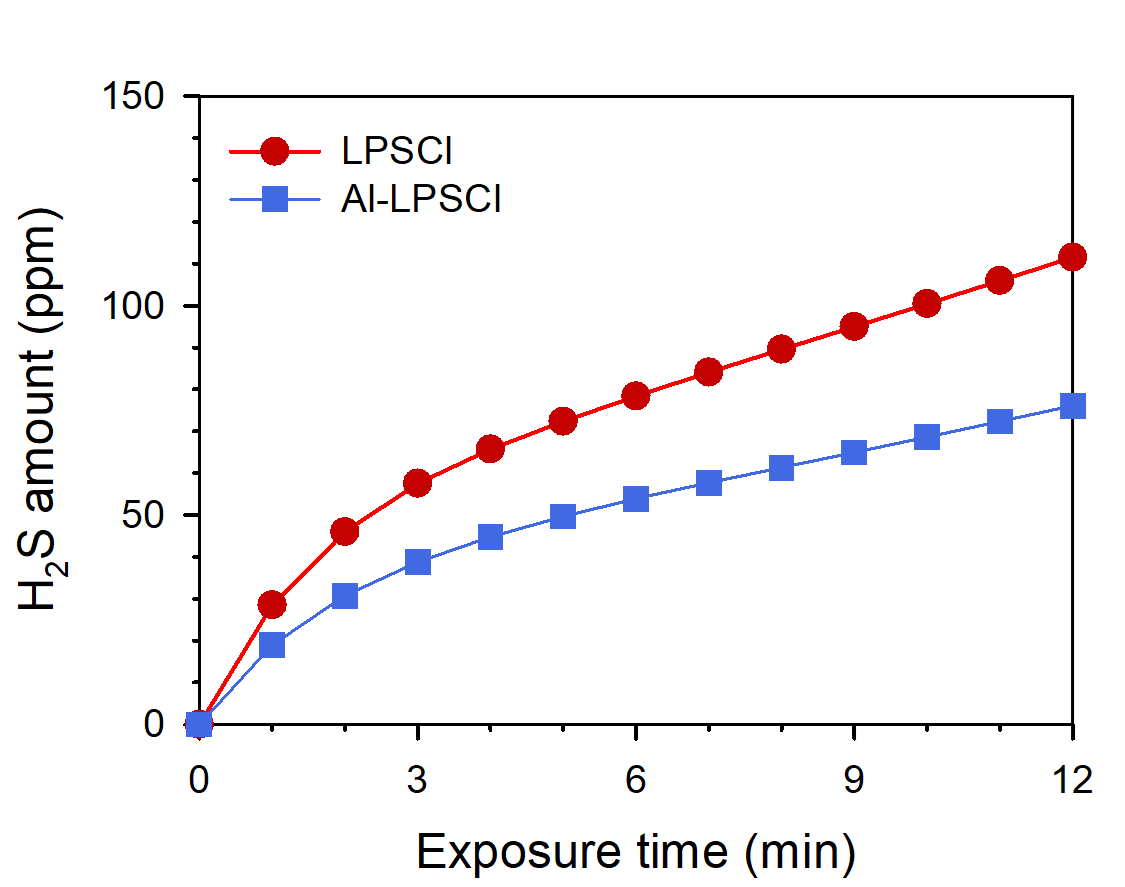


**Figure S8.** H_2_S evolution as a function of time in dry air with a dew point of -40 ^o^C for Li_5.25_Al_0.25_PS_5_Cl compared with that of Li_6_PS_5_Cl.


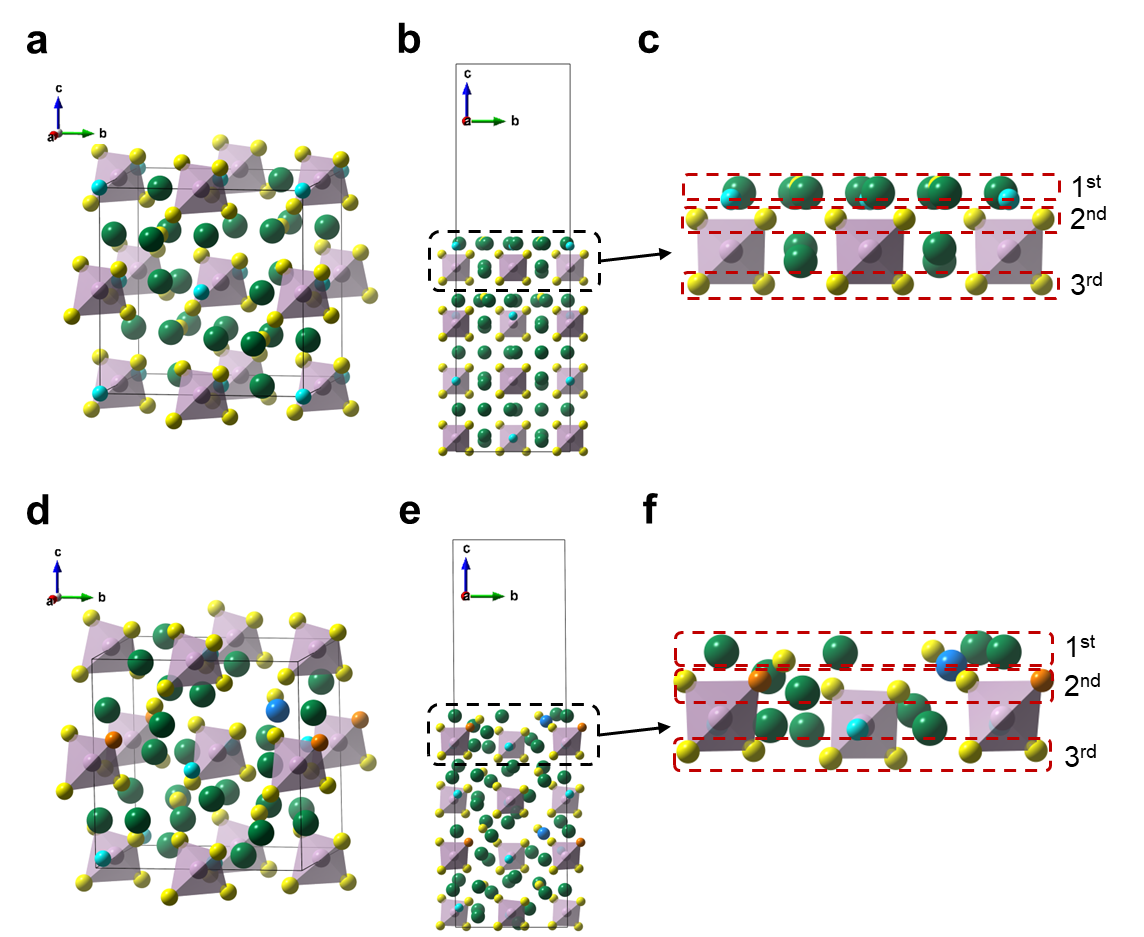


**Figure S9.** Structures of LPSCl and AS-LPSCl. a, d) Bulk crystal structures, b, e) corresponding (001) surface structures, and c, f) enlarged views highlighting the layer arrangements (or orders) of LPSCl (a–c) and AS-LPSCl (d–f) (Li: green, P: purple, S: yellow, Cl: cyan, Al: blue, and Se: orange). The corresponding lattice parameters are listed in Table S5.


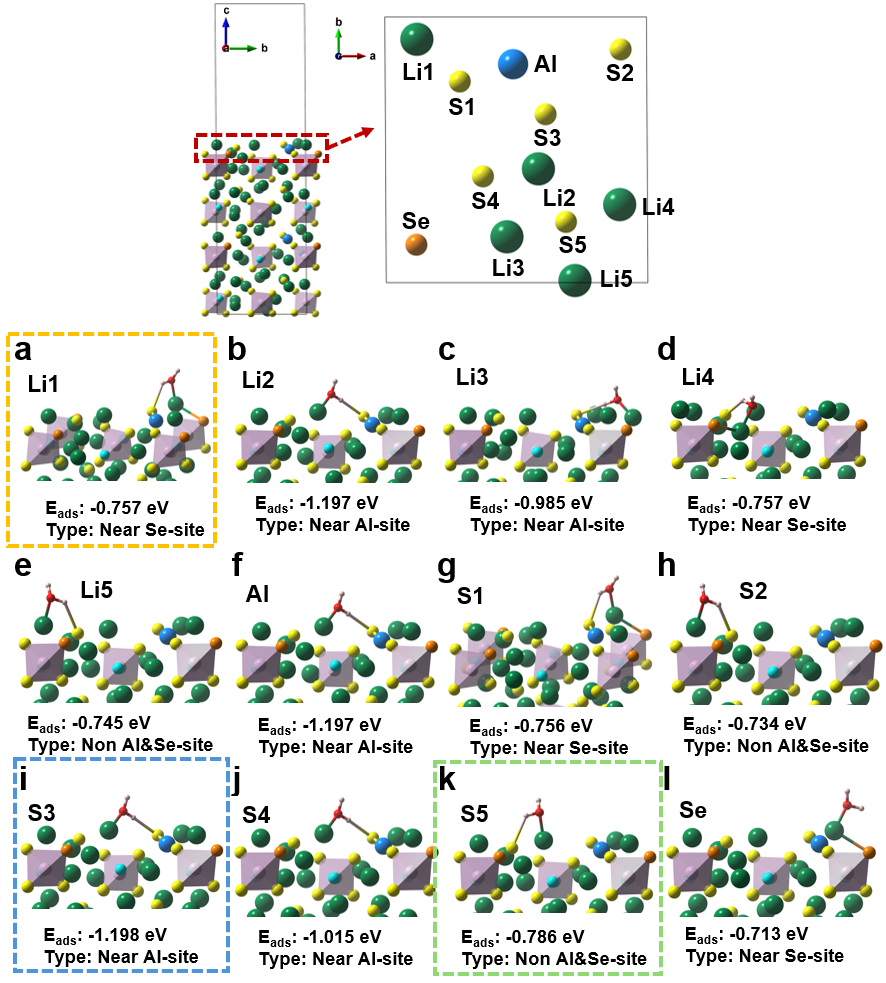


**Figure S10**. Calculated adsorption structures within the AS-LPSCl surface depending on the initial adsorption position of the H_2_O molecule as (a-e) Li sites, f) Al site, (g-k) S sites, and l) Se site, including E_ads_ values and classification into three adsorption types (Li: green, P: purple, S: yellow, Cl: cyan, Al: blue, Se: orange, O: red, and H: pink).


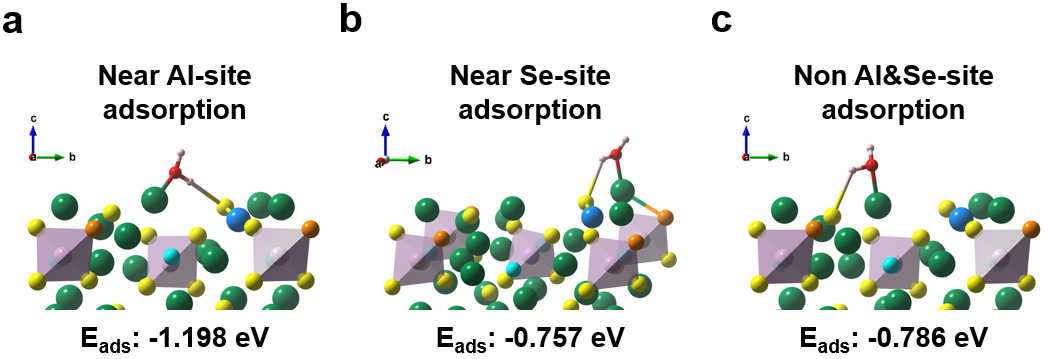


**Figure S11.** Calculated *E_ads_* values for three AS-LPSCl adsorption cases, including adsorption adjacent to a) Al sites, b) Se sites, and c) other sites, regardless of Al and Se dopants (Li: green, P: purple, S: yellow, Cl: cyan, Al: blue, Se: orange, O: red, and H: pink).


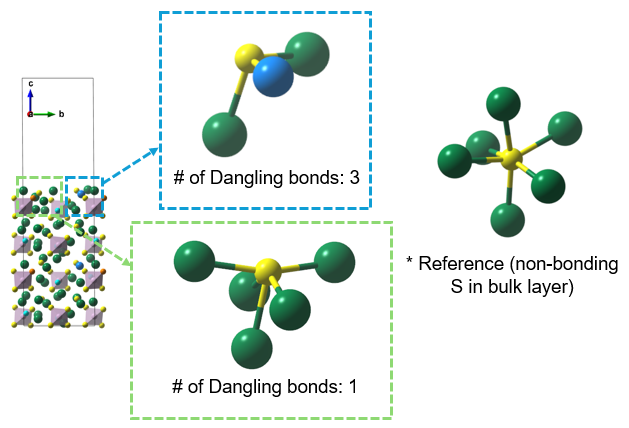


**Figure S12**. Description of the number of dangling bonds on the two cases of surface non-bonding S sites relative to the coordination number of the corresponding atom in the bulk layer.


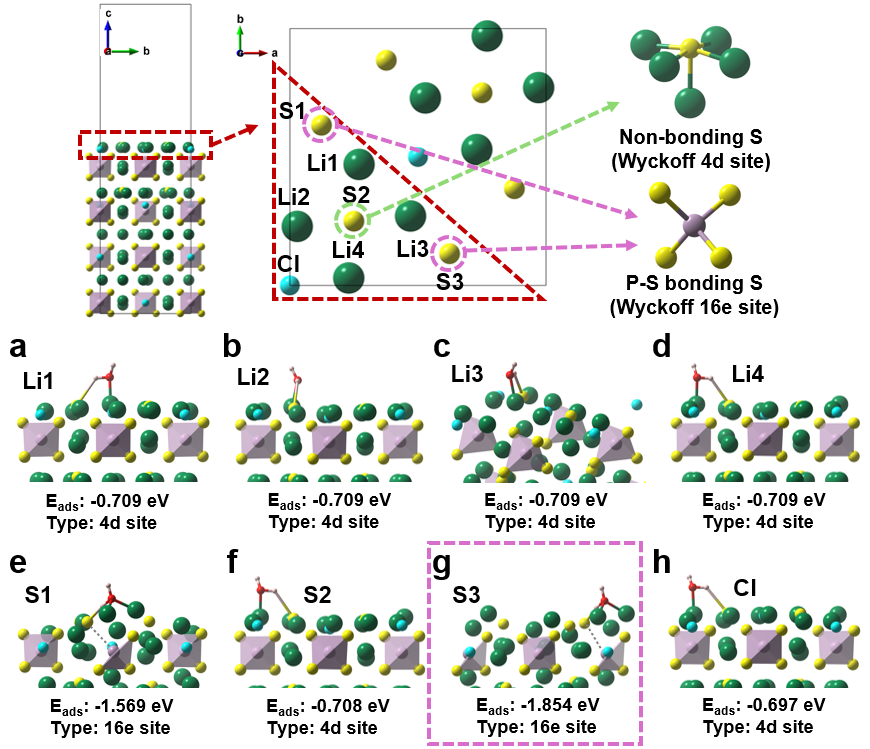


**Figure S13**. Calculated adsorption structures within the LPSCl surface depending on the initial adsorption position of the H_2_O molecule as (a-d) Li sites, (e-g) S sites, and h) Cl site, including *E_ads_* values and classification into two adsorption types, 4d site-dependent adsorption and 16e site-dependent adsorption.


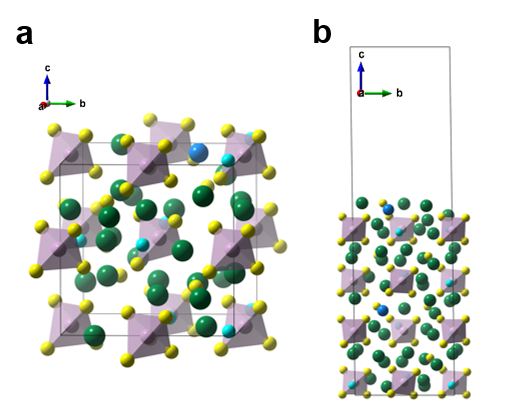


**Figure S14**. Structures of Al-doped LPSCl (Al-LPSCl). a) Bulk crystal structure, and b) corresponding surface structure. The (010) slab was selected for investigating the Al-doping effect due to its direct exposure of the dopant. Given the face-centered symmetry of LPSCl, the (001), (010), and (100) slabs with equivalent surface structures exhibit the same surface energy, which makes the comparison of adsorption stability with the (001) slab of LPSCl valid.


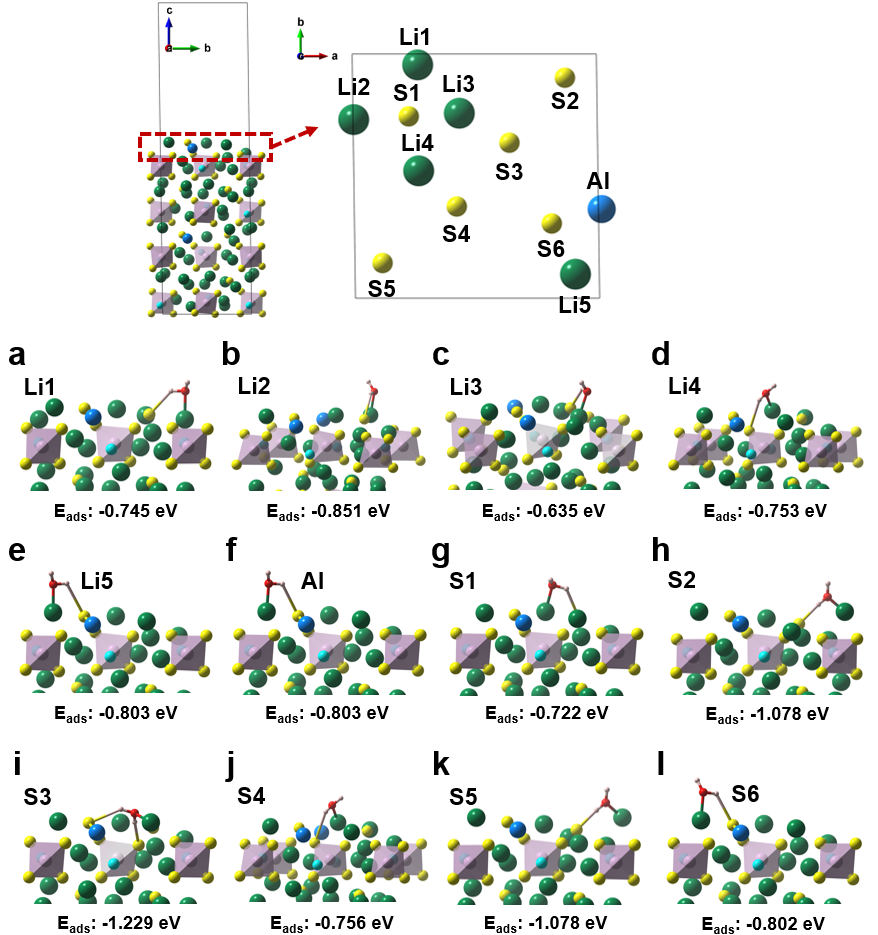


**Figure S15**. Calculated H_2_O adsorption structures within the Al-LPSCl surface as (a-e) Li sites, f) Al site, and (g-l) S sites, including *E_ads_* values.

**
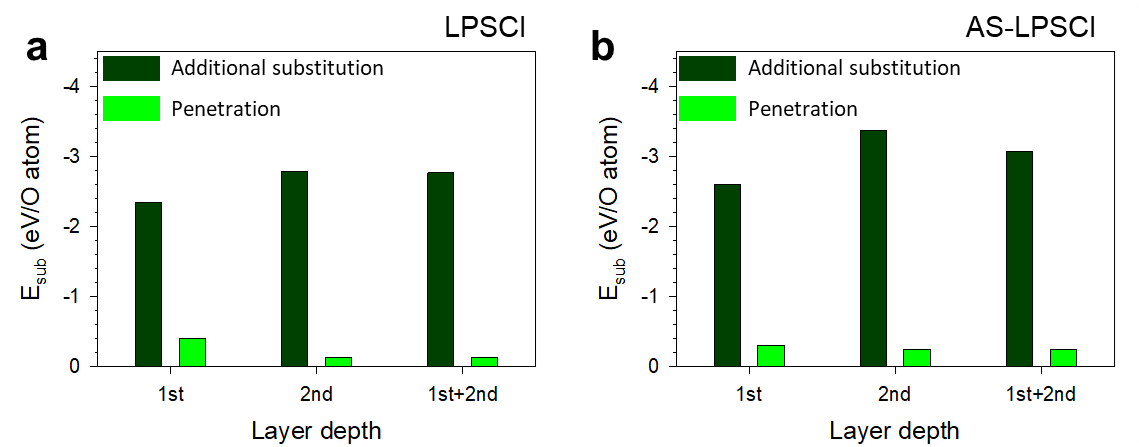
**

**Figure S16.** Comparison of additional surface substitution and penetration based on the S-O substitution energy ($E_{\mathrm{substitution}}$) values for a) LPSCl and b) AS-LPSCl. Both SEs exhibited a lower thermodynamic preference for penetration.


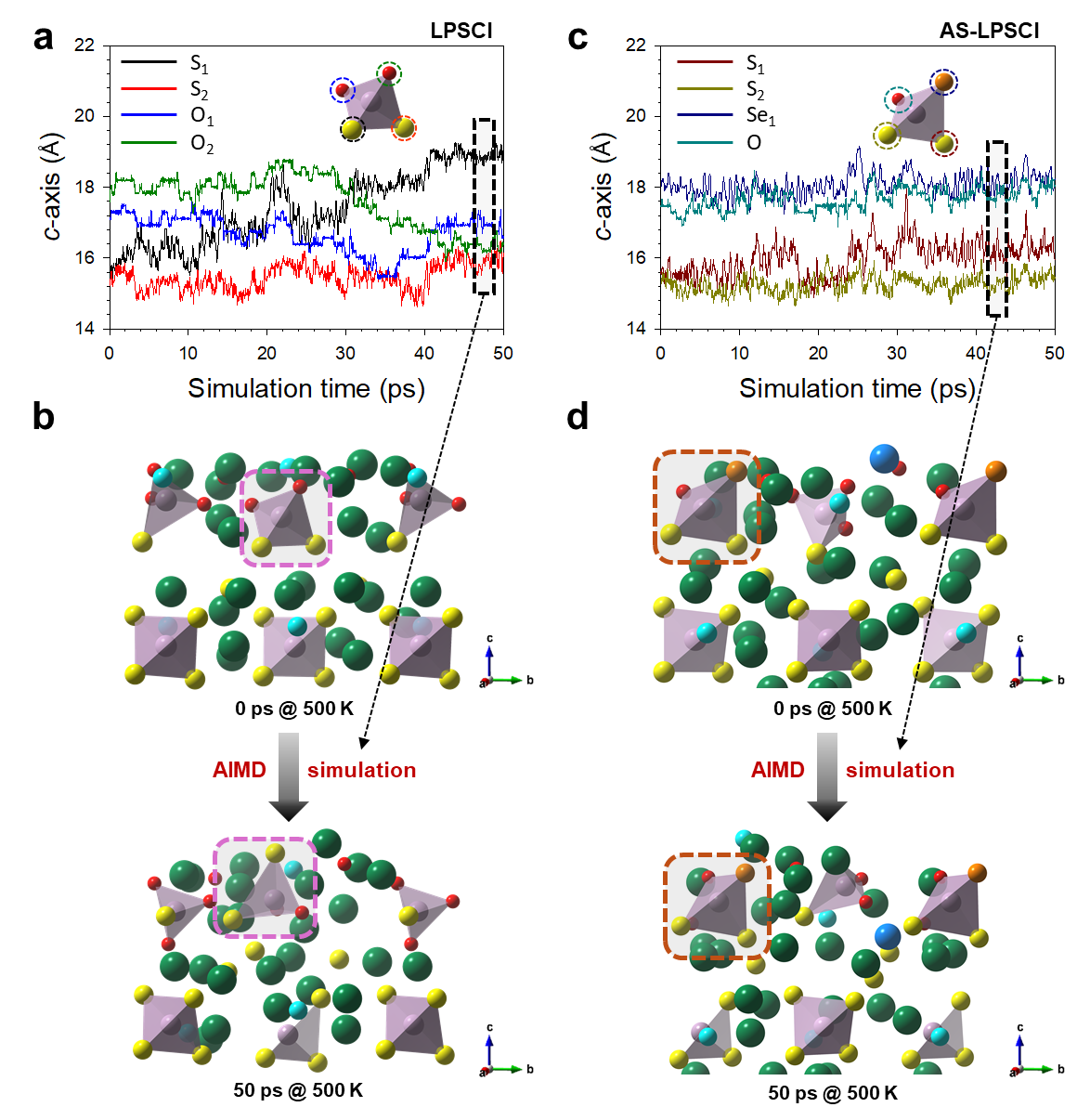


**Figure S17.** a, c) Plots of atomic displacement along the *c*-axis and b, d) corresponding structural changes from AIMD simulations for LPSCl (a, b) and AS-LPSCl (c, d).


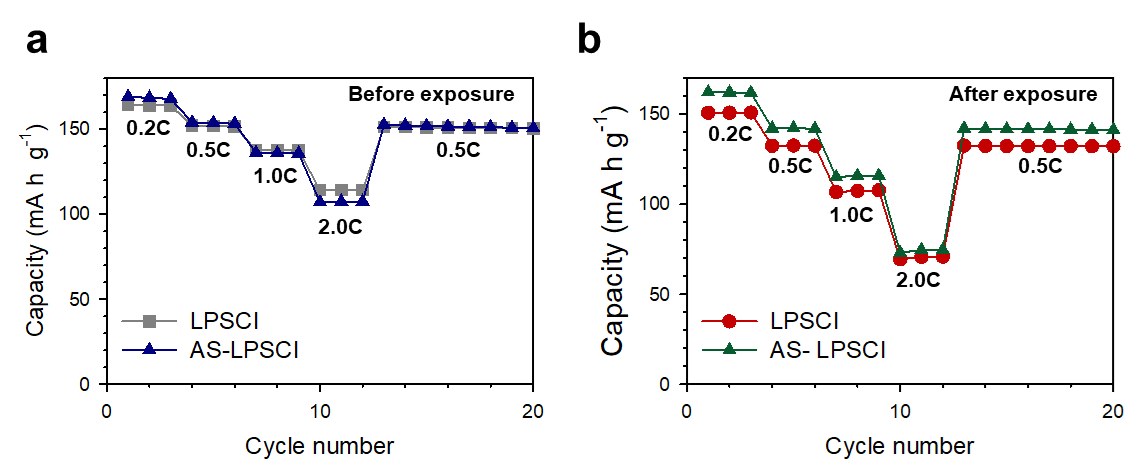


**Figure S18.** Rate capabilities of LPSCl and AS-LPSCl in NCMǀSEǀ(Li-In) cells at 30 °C: a) before and b) after air exposure (at a dew point of −40 °C for 72 h).

2. **Supporting Tables**

**Table S1.** Crystallographic data and Rietveld refinement results for Li_5.850_Al_0.050_PS_4.925_Se_0.075_Cl obtained using HRPD: atomic coordinates, site occupancies, displacement parameters, and reliability factors at room temperature. The corresponding plot is shown in Figure S6.

| **Crystal structure** | | | Cubic | | |  |
| --- | --- | --- | --- | --- | --- | --- |
| **Space group** | | | $F\bar{4}3m$ (216) | | |  |
| **Lattice parameter** | | | a = b = c = 9.84414 | | |  |
| **Atom** | ***x*** | ***y*** | | ***z*** | **B_iso_ (Å^2^)** | **Occ.** |
| Li1 | 0.32000(0) | 0.01820(0) | | 0.67980(0) | 3.000(0) | 6.000(0) |
| Cl4a | 0.00000(0) | 0.00000(0) | | 0.00000(0) | 2.561(50) | 0.385(0) |
| Cl4c | 0.75000(0) | 0.75000(0) | | 0.75000(0) | 3.345(53) | 0.615(0) |
| P1 | 0.50000(0) | 0.50000(0) | | 0.50000(0) | 2.008(45) | 1.000(0) |
| S4c | 0.75000(0) | 0.75000(0) | | 0.75000(0) | 3.345(53) | 0.385(0) |
| S16e | 0.11986(12) | -0.11986(12) | | 0.61986(12) | 3.202(28) | 4.000(0) |
| S4a | 0.00000(0) | 0.00000(0) | | 0.00000(0) | 2.561(50) | 0.615(0) |
| Al | 0.32000(0) | 0.01820(0) | | 0.67980(0) | 3.000(0) | 0.013(0) |
| Se | 0.75000(0) | 0.75000(0) | | 0.75000(0) | 3.345(53) | 0.075(0) |
| *R_p_* = 7.94%, *R_wp_* = 12.2%, *R_exp_* = 10.69% | | | | | | |

**Table S2.** Calculated Meyer–Neldel energies (Δ_0_)^[S1]^ and conduction types for representative Li argyrodites and Li_6-3_*_x_*Al*_x_*PS_5-1.5_*_x_*Se_1.5_*_x_*Cl.

| **Composition** | **Δ_0_ (meV)** | **Type** | **Ref.** |
| --- | --- | --- | --- |
| Li_6-_*_x_*PS_5-x_Cl_1+x_ | 29 | Type 1 | *Angew. Chem. Int. Ed.* **2019**, *58*, 8681–8686. |
| Li_6+_*_x_*Ge*_x_*P_1-_*_x_*S_5_I | 78 | Type 1 | *J. Am. Chem. Soc.* **2018**, *140*, 16330−16339. |
| Li_6-_*_x_*PS_5-x_Br_1+x_ | 28 | Type 1 | *Energy Storage Mater.* **2020**, *30*, 238–249. |
| Li_6-3_*_x_*Al*_x_*PS_5-1.5_*_x_*Se_1.5_*_x_*Cl | 32 | Type 1 | *This work* |

**Supplementary Note 1.** The Meyer–Neldel energy^[S1]^ represents the characteristic energy associated with the compensation effect between the activation energy (E_a_) and the pre-exponential factor (σ_0_) in ionic conduction, as expressed by

$$\sigma= \sigma_{0}exp\left( -\frac{E_{a}}{k_{B}T} \right)$$

$$ln\sigma_{0}= \alpha E_{a}+\beta,$$

$\Delta_{0}= \frac{1}{\alpha}$.

Physically, Δ_0_ quantifies how changes in lattice vibrations, local structural dynamics, or ion–lattice interactions compensate for variations in the migration barrier (E_a_). A large Δ_0_ indicates that the ionic motion is strongly coupled with thermally activated lattice excitations, whereas a small Δ_0_ implies that the hopping process is less sensitive to such lattice effects.^[S1]^ Depending on the relative magnitude of Δ_0_ with respect to the thermal energy (k_B_T ≈ 26 meV at 300 K), ionic conductors can be classified into three types:

- Type 1 (Δ_0_ >$k_{B}T$): conductivity increases as Eₐ decreases.
- Type 2 (Δ_0_ <$k_{B}T$): conductivity increases with increasing Eₐ due to overcompensation in$\sigma_{0}$.
- Type 3 (Δ_0_ ≈$k_{B}T$): conductivity is nearly independent of E_a_.

As widely reported—and as noted by the reviewer—Li argyrodites, in which Li vacancies and Cl/X site disorder facilitate Li⁺ transport, belong to Type 1 (Δ_0_ > 26 meV), showing higher conductivity with lower activation energy (Table S2). Similarly, Li_6-3_*_x_*Al*_x_*PS_5-1.5_*_x_*Se_1.5_*_x_*Cl exhibits a Δ_0_ value of 32 meV, indicating that it also falls into the Type 1 category, where a decrease in E_a_ leads to enhanced conductivity. Therefore, it can be concluded that the fundamental ionic transport mechanism of the material itself remains unchanged.

However, as pointed out by the reviewer, there are certain composition ranges in which both the ionic conductivity and the activation energy increase simultaneously (**Figure 2c**). This occurs because the pre-exponential factor (σ_0_) varies concurrently with E_a_; the conductivity gain resulting from a lower E_a_ is counterbalanced by a reduction in σ_0_. As a result, in narrow composition windows, it may appear that samples with higher conductivity also exhibit larger E_a_ — a local compensation phenomenon rather than evidence of a fundamental change in the Li⁺ transport pathway or mechanism.

[S1] Y. Gao, N. Li, Y. Wu, W. Yang, S.-H. Bo, *Adv. Energy Mater.* **2021**, *11*, 2100325.

**Table S3.** Bulk resistance (R_1_), grain boundary resistance (R_2_), and ionic conductivity of pristine LPSCl and AS-LPSCl after air exposure for 0, 5, and 72 h at a dew point of −40 °C. The equivalent circuit model shown in **Figure 3b** was used for fitting.

| **Sample** | **Exposure time (h)** | **R_1_ (bulk) (Ω)** | **R_2_ (grain boundary) (Ω)** |
| --- | --- | --- | --- |
| LPSCl | 0 | 75.1 | 3.4 |
|  | 5 | 88.7 | 83.2 |
|  | 72 | 83.2 | 2760 |
| AS-LPSCl | 0 | 63.8 | 1.9 |
|  | 5 | 62.6 | 16.0 |
|  | 72 | 71.0 | 1254 |

**Table S4.** Relative surface bonding ratios of LPSCl and AS–LPSCl before and after air exposure, as determined via XPS (**Figure 3e**).

| **Sample** | **Bonding** | **Ratio (%)** | |
| --- | --- | --- | --- |
|  |  | **Before exposure** | **After exposure** |
| LPSCl | P-S | 93.4 | 86.0 |
|  | P-O | 6.6 | 14.0 |
| AS-LPSCl | P-S | 88.4 | 84.0 |
|  | P-O | 1.8 | 5.1 |
|  | P-Se | 9.8 | 10.9 |

**Table S5.** Lattice parameters for the calculated slab structures of LPSCl, AS-LPSCl, and Al-LPSCl compared with the bulk structures.

| **Structure** | | **Lattice parameter (Å)** | | |
| --- | --- | --- | --- | --- |
|  |  | ***a*** | ***b*** | ***c*** |
| LPSCl | Bulk | 10.256 | 10.256 | 10.256 |
|  | Slab | 10.256 | 10.256 | 34.767 |
| AS-LPSCl | Bulk | 9.902 | 10.044 | 10.057 |
|  | Slab | 9.902 | 10.044 | 34.770 |
| Al-LPSCl | Bulk | 9.945 | 10.088 | 9.913 |
|  | Slab | 9.913 | 9.945 | 34.764 |

**Table S6.** Discharge capacities of NCM half cells with LPSCl or AS-LPSCl electrodes before and after air exposure at various C rates, together with the corresponding retention ratios (B/A). These results highlight the superior capacity retention of AS-LPSCl compared with that of pristine LPSCl upon air exposure.

|  | **Discharge capacity before air exposure (A)**  **(mAh g^-1^)** | | **Discharge capacity after air exposure (B)**  **(mAh g^-1^)** | | **Retention Ratio (B/A)**  **(%)** | |
| --- | --- | --- | --- | --- | --- | --- |
|  | **LPSCl** | **AS-LPSCl** | **LPSCl** | **AS-LPSCl** | **LPSCl** | **AS-LPSCl** |
| 0.2C | 164.3 | 169.0 | 150.7 | 162.2 | 91.7 | 96.0 |
| 0.5C | 152.0 | 154.0 | 132.4 | 142.2 | 87.1 | 92.3 |
| 1.0C | 137.6 | 136.1 | 106.8 | 115.1 | 77.6 | 84.6 |
| 2.0C | 114.2 | 107.1 | 69.4 | 73.1 | 60.8 | 68.2 |
